# Enhancing plant defense using rhizobacteria in processing tomatoes: a bioprospecting approach to overcoming Early Blight and *Alternaria* toxins

**DOI:** 10.3389/fmicb.2023.1221633

**Published:** 2023-08-04

**Authors:** Gabriele Bellotti, Maria Chiara Guerrieri, Paola Giorni, Giulia Bulla, Andrea Fiorini, Terenzio Bertuzzi, Maria Elena Antinori, Edoardo Puglisi

**Affiliations:** ^1^Department for Sustainable Food Process, Università Cattolica del Sacro Cuore, Piacenza, Italy; ^2^Department of Sustainable Crop Production, Università Cattolica del Sacro Cuore, Piacenza, Italy; ^3^Department of Animal Science, Food and Nutrition, Università Cattolica del Sacro Cuore, Piacenza, Italy

**Keywords:** biocontrol agents, microbial-based biopesticides, PGPR, *Bacillus*, *Alternaria*, mycotoxins, tomato, bacterial-fungal interactions

## Abstract

Plant growth-promoting rhizobacteria (PGPR) with antagonistic activity toward plant pathogenic fungi are valuable candidates for the development of novel plant protection products based on biocontrol activity. The very first step in the formulation of such products is to screen the potential effectiveness of the selected microorganism(s). In this study, non-pathogenic rhizobacteria were isolated from the rhizosphere of tomato plants and evaluated for their biocontrol activity against three species of mycotoxin-producing *Alternaria*. The assessment of their biocontrol potential involved investigating both fungal biomass and *Alternaria* toxin reduction. A ranking system developed allowed for the identification of the 12 best-performing strains among the initial 85 isolates. Several rhizobacteria showed a significant reduction in fungal biomass (up to 76%) and/or mycotoxin production (up to 99.7%). Moreover, the same isolates also demonstrated plant growth-promoting (PGP) traits such as siderophore or IAA production, inorganic phosphate solubilization, and nitrogen fixation, confirming the multifaceted properties of PGPRs. Bacillus species, particularly *B. amyloliquefaciens* and two strains of *B. subtilis*, showed the highest efficacy in reducing fungal biomass and were also effective in lowering mycotoxin production. Isolates such as *Enterobacter ludwigii, Enterobacter asburiae, Serratia nematodiphila, Pantoea agglomerans*, and *Kosakonia cowanii* showed moderate efficacy. Results suggest that by leveraging the diverse capabilities of different microbial strains, a consortium-based approach would provide a broader spectrum of effectiveness, thereby signaling a more encouraging resolution for sustainable agriculture and addressing the multifaceted nature of crop-related biotic challenges.

## 1. Introduction

Tomato (*Solanum lycopersicum* L.) is the second most important plant grown worldwide after potatoes, and in 2020, global production reached approximately 187.5 million tons^1^, surpassing previous years' productions. Italy is the major EU tomato supplier (Iotti and Bonazzi, 2018).

Because of their soft epidermis and high water content, tomatoes are known to be highly susceptible to fungal colonization (Moss, 1984). One of the most common fungi that infects tomato plants and fruits is *Alternaria*, with the most frequently reported being *A. solani, A. alternata*, and *A. tenuissima* (Garganese et al., 2018; Sanzani et al., 2019). These fungi are considered responsible for the Early Blight (EB) disease and severe damage to tomato plants when environmental conditions are favorable, with symptoms on foliage, stems, and fruits, resulting in yield losses ranging from 35 to 78% (Parvin et al., 2021). In addition, they produce different kinds of mycotoxins generally referred to as *Alternaria* toxins, of which the most important in terms of human exposure and toxicity are alternariol (AOH), alternariol monomethyl ether (AME), tenuazonic acid (TeA), and tentoxin (TEN) (Aichinger et al., 2021; Lin et al., 2023). As stated by the European Food Safety Authority (EFSA) in the latest scientific opinion on *Alternaria* toxin, tomato and its derivates largely impact consumer exposure (EFSA et al., 2016). Indeed, on 5 April 2022, the EU emanated the Recommendation (EU) 2022/553 which supplemented the Regulation (EU) 1881/2006 taking *Alternaria* toxins into consideration and setting limits in foodstuffs of 2 μg/kg for AOH; moreover, in 2022, EFSA prepared some recommendations for *Alternaria* toxins in all the processed products of tomato (Publications Office of the European Union., 2022).

Currently, no *Alternaria*-resistant tomato varieties are available (Ray et al., 2015) and, while much progress has been made in the Italian farming system with the adoption of Integrated Pest Management (IPM) practices (Ciampitti and Cavagna, 2014), the use of agrochemicals remains one of the best options to protect tomato production. At the same time, consumer demand for organic products has led to an increase in Italian farms transitioning to organic production systems, which typically allow for the use of copper-based fungicides as the sole means of plant protection. It is well-established that the overuse of chemicals for plant protection can lead to environmental disturbances, bioaccumulation in food, environmental pollution, harm to beneficial non-target organisms, and the development of fungicide-resistant strains of plant pathogens. Although to a much lower extent, the same phenomena have also been observed for copper-based fungicides (Shoaib et al., 2015; Tamm et al., 2022). These downsides highlight the importance of introducing novel and effective broad-spectrum plant protection products to be included in IPM programs, so as to lower the use of fungicides while maximizing sustainability and respecting the environment. Plant growth-promoting rhizobacteria (PGPR) represent a promising alternative for sustainable crop management. Their role in sustainable agriculture is mainly known for establishing plant–microbe interaction belowground, in association with plant roots, where they can boost key physiological plant processes that contribute to setting up plants in a better nutritional state and to better cope with environmental stresses, thus promoting plant growth and development (Glick, 2012). On top of that, many scientific studies demonstrate how PGPR can be effective for the biological control of major fungal pathogens due to the production of antimicrobial compounds, lytic enzymes, and the competition of nutrients and space (Singh et al., 2023). Moreover, PGPR can also enhance plant defense indirectly, through an intricate mechanism known as induced systemic resistance (ISR) (Pieterse et al., 2014), showing PGPR multiple modes of action for the sustainment of plant defense. Studies focusing on the biological control of EB in tomato crops reported evidence that PGPRs can be effectively used to control EB incidence in tomato plant cultivations in both greenhouse (Panebianco et al., 2022) and in field experimentation (Karthika et al., 2020). These studies highlight several characteristics of *Bacillus* species that make them desirable biocontrol agents: (i) they compete against pathogenic microorganisms for space and nutrients, (ii) produce a wide variety of antimicrobial compounds, (iii) induce ISR, and (iv) show mycoparasitism ability (Legein et al., 2020). Moreover, they can degrade/inhibit the production of toxic compounds as demonstrated by Jia et al. (2023) where *B. amyloliquefaciens* drastically reduced the production of TeA by *A. alternata*.

In light of their ability to promote plant growth and suppress pathogenic fungi, PGPRs represent a promising avenue for exploration in the context of IPM, organic, or traditional production. Additionally, their ability to inhibit the production of toxic fungal metabolites that could enter the food chain and pose a risk to consumers further underscores their potential as a viable solution to reducing reliance on traditional pesticides in agriculture.

In this study, we explored the root-associated rhizobacteria of tomato plants that can be cultured and evaluated their ability to control three mycotoxigenic strains of *Alternaria*. We measured the biocontrol effect by assessing the reduction in fungal growth and inhibition of the production of the main *Alternaria* toxins. We hypothesized that the interaction between PGPR and pathogenic fungi *in vitro* could not only limit fungal growth but also modify the production of toxic metabolites such as *Alternaria* toxins. Additionally, we evaluated the biostimulant activity of the best-performing strains, demonstrating the diverse applications that PGPR can have in sustainable agriculture. To ensure that our study focused on the most promising isolates, a ranking method was developed during the screening process. This method allowed us to select the best-performing isolates from a wide variety of options, which will be considered for future *in-planta* experiments.

## 2. Materials and methods

### 2.1. Isolation of putative growth-promoting rhizospheric and endophytic bacteria

Roots of healthy tomato plants were collected in July 2020 in a commercial field situated in Gabbioneta-Binanuova (45 12′03.0” N 10 12′27.8” E), Cremona, Po Valley (Northern Italy). Tomato plants were carefully uprooted from the soil, packaged in sterile polybags, cooled at 4°C, and immediately shipped to the laboratory for the isolation of both rhizospheric and endophytic bacteria.

The isolation of bacteria from the rhizosphere was performed according to Barillot et al. (2013). Briefly, bulk soil was removed by vigorously shaking plants by hand, paying attention to the roots' integrity as long as the loosely adhering soil was completely removed. Afterward, the root system was washed with sterile 0.9% saline solution and mixed with Tween 80 (0.01% v/v), and the mixture was incubated at 25°C for 90 min with shaking at 180 rpm. The resulting suspensions were serially diluted (10^−8^), and 0.1 mL aliquots were spread in triplicate on LB (Luria–Bertani) (Oxoid, Basingstoke, UK) for each dilution. To prevent any fungal growth, plates were amended with cycloheximide [0,1%, Sigma-Aldrich, St. Louis (MO), USA] and incubated at 30°C for 24–72 h. Morphologically distinct bacterial colonies of rhizosphere isolates were selected and purified by repeated streaking on LB agar. Isolates were temporarily cryopreserved at −20°C in 20% glycerol for further studies.

For endophytic isolation, the collected roots were thoroughly washed under running tap water to remove rhizosphere soil and then sterilized by sequential immersion in 70% ethanol for 1 min and in household bleach (sodium hypochlorite) diluted in sterilized water (1:1) for 2 min, and finally rinsed five times in sterile distilled water to remove surface sterilization agents. Ten grams of root were transversally cut into small pieces of approximately 0.5 to 1 cm using a sterile single-use scalpel blade, placed in sterile physiological water, and incubated overnight at 25°C with shaking at 180 rpm. Serial dilutions from 10^−1^ to 10^−5^ were prepared, and 0.1 mL of aliquots were spread onto LB agar amended with cycloheximide, in triplicates, and the plates were incubated for 24–72 h at 30°C. To verify the efficacy of surface sterilization of the roots, a root segment after the last rinse was placed on the LB medium and incubated (Ambrosini and Passaglia, 2017). Morphologically distinct bacterial colonies were selected, and pure cultures were cryopreserved in a 20 % glycerol solution at −20°C.

### 2.2. Rep-PCR dereplication of isolates

Genomic DNA from rhizospheric and endophytic isolates was extracted using the MicroLYSIS^®^ Plus Kit (Microzone, Haywards Heath, UK). According to Sadowsky and Hur (1998), the genetic diversity among the isolates obtained was assessed by means of the rep-PCR genotyping technique, using GTG5 (5′-GTGGTGGTGGTGGTG-3′) as a primer. PCR products were checked by electrophoresis in a 2.5% agarose gel, and the profiles were visualized with the software Image Lab (Bio-Rad). The comparative analysis of the resulting fingerprints was performed using the software Geljv.2.0 (Heras et al., 2015).

### 2.3. Identification of unique isolates through 16S rRNA gene sequencing

PCR amplification of 16S rDNA, from representative strains of each rep-PCR profile, was carried out using the universal primers P1 (5′-GCGGCGTGCCTAATACATGC-3′) and P6 (5′-CTACGGCTACCTTGTTACGA-3′) as described by Di Cello and Fani (1996). The PCR reaction mixture and conditions were the same as described by Guerrieri et al. (2020). Sanger sequencing of PCR products was carried out at GATC Biotech (Ebersberg, Germany). The obtained 16S ribosomal DNA sequences were compared with others in the GenBank database, using RDP (Ribosomal database project) at http://rdp.cme.msu.edu/and confirmed via NCBI-BLAST server, at https://blast.ncbi.nlm.nih.gov/Blast.cgi.

### 2.4. *In vitro* screening of rhizospheric and endophytic bacterial isolates for antagonism against *Alternaria* spp.

Rhizosphere and endosphere bacterial isolates were screened for their antagonism in dual culture assay. One strain of *A. alternata* (CBS 118814), one strain of *A. solani* (CBS 109157), and one strain of *A. tenuissima* (CBS 117.44), obtained from the Westerdijk Fungal Biodiversity Institute (Utrecht, the Netherlands), able to produce mycotoxins (TeA, AOH, AME, and TEN) were used for the dual plate inoculation experiment. The fungal strains were singularly inoculated on Petri dishes (Ø 9 cm) with potato dextrose agar (PDA, BioLife, Milano, Italy) and incubated at 25°C for 7 days (12 h light/12 h dark photoperiod). At the end of incubation, developed fungal colonies were used to *in vitro* test the antagonistic ability of the different rhizobacteria strains, performing a dual plate confrontation assay. Bacterial isolates were cultured in 10 mL tubes containing 5 mL of LB broth, incubated overnight at 30°C. Cell density was standardized using a McFarland barium sulfate standard 1, corresponding to 3 × 10^8^ CFU/mL, by adding sterile 0.9% saline solution until the appropriate cell density was reached (Zapata and Ramirez-Arcos, 2015). Agar disks were homogeneously cut from the developed fungal colonies using a sterile cork borer (Ø 4 mm) and placed at the center of a Petri dish (Ø 9 cm) containing PDA (BioLife, Milano, Italy). Standardized bacterial suspensions obtained from each isolate were streaked aseptically, parallel to the fungus at 15–20 mm on both sides of the fungal plug (Anith et al., 2021). Petri dishes used for the dual plate assay were incubated at 25°C, and fungal growth was measured (recording the two perpendicular diameters of the fungal colony after 7 and 14 days) and used to calculate the fungal growth reduction in comparison to the growth obtained by each fungal strain cultivated alone. At the end of incubation (14 days), the entire content of the Petri dishes was used for mycotoxin quantification.

### 2.5. Analysis and determination of *Alternaria* toxins

Quantification of Alternaria mycotoxins was carried out according to the method reported by Bertuzzi et al. (2021). Briefly, AOH, AME, TeA, and TEN were simultaneously extracted from a 25 g sample with 100 mL of water:acetonitrile 20+80 mixture (v/v), using a rotary-shaking stirrer for 45 min. After filtration, dilution, and purification on an OASIS HLB column (6cc, 500 mg Waters Corporation, Bedford, MA, USA), *Alternaria* toxins were eluted in a graduated vial using 6 mL of acetonitrile and concentrated to 2 mL under a gentle stream of nitrogen. After dilution (1+2) with the water:acetonitrile 70+30 mixture (v/v), the final extract was injected (20 μl) into the LC-MS/MS system (Thermo Fisher Scientific, San Jose, CA, USA) and a PAL 1.3.1 sampling system (CTC Analytics AG, Zwingen, Switzerland). The *Alternaria* toxins were separated on a BetaSil RP-18 column (5 μm particle size, 150 × 2.1 mm, Thermo Fisher) by gradient elution with acetonitrile and water (both acidified with 0.2% formic acid) from 35:65 to 75:25 in 5 min, then isocratic for 2 min at a flow rate of 0.2 mL min-1. The ionization was carried out using an ESI interface (Thermo-Fisher) in positive mode as follows: spray capillary voltage 4.5 kV, sheath, and auxiliary gas 35 and 12 psi, respectively, skimmer offset 6 kV, temperature of the heated capillary 350°C. The selected ions were: 128, 185, and 213 m/z (35 V) for AOH, 128, 184 m/z (38 V), and 258 m/z (30 V) for AME, 125, 139, and 153 m/z (16 V) for TeA, and 132 m/z (37 V), 135, and 312 m/z (25 V) for TEN, respectively. Quantitative determination was performed by LC_Quan 2.0 software (Thermo_Fischer). Even for mycotoxins, percentages of reduction for each mycotoxin considered were calculated in comparison with mycotoxin production obtained if the fungus was cultivated alone.

A preliminary screening was carried out on a total of 92 isolates (data not shown), considering single measurements of the abovementioned parameters of mycotoxin and fungal biomass, while replicate measurements (quintuplicates and triplicates for fungal growth and mycotoxin production, respectively) were prepared on the selected set of isolates that showed at least 40% inhibition of at least one parameter in the preliminary test.

### 2.6. *In vitro* assessment of PGP traits

Inoculations were made from McFarland 1.0 standardized bacterial cultures as described in the dual culture assay. For a relative estimation of phosphate-solubilizing properties, selected rhizospheric and endophytic isolates were inoculated in NBRIP broth supplemented with 0.025 mg/mL of bromophenol blue (BPB), designated as NBRIP-BPB, and incubated for 7 days at 30°C. Optical density was taken at 600 nm by using a UV/visible spectrophotometer (Mehta and Nautiyal, 2001).

The phytohormone indole acetic acid (IAA) production was estimated using the Salkowski reagent (1 mL of 0.5 M FeCl_3_ in 50 mL of 35% HClO_4_) following the protocol proposed by Harikrishnan et al. (2014). Bacterial isolates were inoculated in LB medium supplemented with the precursor L-tryptophan (0.1%) (Sigma-Aldrich, St. Louis, MO, USA) and incubated at 30°C for 7 days. After the incubation, the cultures were centrifuged at 4°C for 10 min (10.000) rpm. The supernatant was mixed with Salkowski (1:2 v:v) and incubated in the dark for 1 h. The development of red color indicated the presence of IAA. The optical density was taken at 540 nm by using a UV/visible spectrophotometer. A standard curve of IAA was used to measure the concentration of IAA produced.

The strains were also quantitatively assessed for siderophores production using the Chrome Azurol Sulphonate (CAS) reagent (Schwyn and Neilands, 1987). According to Dimkpa (2016), isolates were inoculated in a siderophore-inducing medium (SIM) and incubated for 7 days at 30°C. Bacterial cultures were centrifuged at 10.000 rpm for 10 min, and the supernatant was mixed with the CAS solution (1:1 v:v). After 1 h of incubation at room temperature, absorbance was measured to estimate the loss of blue color to orange/yellow.

The nitrogen fixation activity was measured with a slightly modified plate assay method proposed by Tang et al. (2020) by spot inoculation of the strain on N-free malate (Nfb) medium, in quadruplicate, and incubated at 30°C. After 24–48 h of incubation, plates were observed for the development of a blue halo around the colony due to the formation of ammonium (NH_4_) *via* nitrogen fixation metabolic activity. The halos were evaluated by assigning levels as follows: isolates without a halo were considered non-nitrogen fixing or level 1 nitrogen fixator, isolates with a halo bigger than 0 cm up to 1.00 cm were considered level 2 nitrogen fixator, isolates with a halo bigger than 1.00 cm up to 2.00 cm were considered level 3 nitrogen fixator, isolates with a halo bigger than 2.00 cm up to 3.00 cm were considered level 4 nitrogen fixator, and isolates with a halo bigger than 3.00 cm were considered level 5 nitrogen fixator.

### 2.7. Data analysis and ranking system

Dual plate results were expressed as means ± standard deviation. Differences between means were determined by independent sample *t-*tests, conducted on Rstudio (v 2022.12.0.353).

To facilitate a proper selection for further tests, among the best-performing rhizobacterial isolates, a ranking system was developed to rank strains according to their ability to impact *Alternaria* spp. growth and *Alternaria* toxins production. For each isolate, a score was assigned according to its ability to reduce the growth of the three *Alternaria* species considered and inhibit *Alternaria* toxin production. The sum of the scores assigned for each of the assayed properties, for each *Alternaria* species, was considered a global antifungal ability score and was used to rank the isolates from the best performing to the less performing. The ranking values were assigned to each isolate in a range from 0 to 2 depending on the reduction shown in the dual plate assay. On the other hand, a ranking with a value in the range −1 to −3 was assigned to isolates that either resulted unable to reduce fungal growth or, in some cases, increased mycotoxin production. Ranking values assigned according to inhibition are reported in Supplementary Table S1. In the end, each bacterial strain had a rank coming from five different aspects: fungal growth reduction, TeA, AOH, AME, and TEN reductions.

Barplots were created using the barplot() function in RStudio, supplemented with the tidyverse package, including data on the best-performing strains, to visualize differences in fungal growth and mycotoxin production among them. The height of each bar represented the mean of fungal growth (mm) or mycotoxin production (μg) for each strain, and the error bars indicated the standard deviation. *P*-values lower than 0.05 were considered statistically significant and were indicated on the barplots using asterisks according to the level of significance. Principal component analysis (PCA) was conducted to explore the multivariate relationships among the variables considered in the experiment. The PCA was performed using the pca () function in Rstudio supplemented with the FactoMineR package, and the standardized variables were used as input.

## 3. Results and discussion

### 3.1. Isolation of rhizospheric and endophytic bacteria, and molecular characterization

A total of 106 bacteria were successfully collected from the rhizosphere of the tomato plant *Solanum lycopersicum* L., comprising 85 rhizospheric bacteria and 21 endophytic bacteria. Repetitive-element band pattern analysis using rep-PCR allowed for the identification and exclusion of 14 isolates that showed identical patterns to others (data not shown). Subsequent taxonomic identification using BLASTN analysis of 16S rDNA sequences revealed the full list of isolated unique strains, as reported in Supplementary Table S2.

Taxonomic assignment based on 16S rDNA sequences showed that some species identify as pathogens or opportunistic pathogens. Further tests were not conducted on the following isolates: *Rhizobium radiobacter* TR73 (formerly known as *Agrobacterium tumefaciens*), *Tsukamurella pulmonis* TR78, *Xanthomonas axonopodis* TR81, *Xanthomonas hydrangea* TR87, and K*luyvera cryocrescens* TE95, and all isolates belonging to the *Bacillus cereus* s.l. clade TR35, TR37, and TR53 were excluded from *in vitro* tests. However, some isolates such as *Stenotrophomonas maltophilia* TR16, TR33, *Serratia marcescens* TR117, *Pseudomonas brassicacearum* TR88, and *Pantoea agglomerans* TE109, TE116, and isolates belonging to the *Enterobacter cloacae* complex TR61, TE99, TE100, and TE103 were considered for *in vitro* experimentation. Despite occasional reports of opportunistic pathogenicity, these species are often reported for their plant growth promotion and biocontrol properties.

After molecular typing and exclusion of pathogens, the final number of isolates included for *in vitro* assays was reduced to 73 rhizospheric isolates and 12 endophytic isolates, totaling 85 rhizobacteria.

### 3.2. Reduction of *Alternaria* fungal growth and mycotoxin production

In the preliminary screening (data not shown), 85 rhizobacteria were tested for their antifungal activity against three species of *Alternaria* recording the fungal growth and changes in the four analyzed *Alternaria* toxins. More than 50% of the tested strains showed antifungal activity and/or reduced toxin production. Consequently, 45 bacterial strains were selected for a second screening with replicates, and the results are presented in Table 1, while the % difference in growth (mm) and mycotoxin (μg) between each strain and the three *Alternaria* species is reported in Supplementary Table S3.

**Table 1:** Rhizobacteria dual plate assay results vs. *A. alternata, A. solani*, and *A. tenuissima* of the selected 45 isolates.

**Strain**	**Taxonomy**	***A. alternata***					***A. solani***				***A. tenuissima***				
		**Fungal growth (mm)**	**TeA (**μ**g)**	**AOH (**μ**g)**	**AME (**μ**g)**	**TEN (**μ**g)**	**Fungal growth (mm)**	**AOH (**μ**g)**	**AME (**μ**g)**	**TEN (**μ**g)**	**Fungal growth (mm)**	**TeA (**μ**g)**	**AOH (**μ**g)**	**AME (**μ**g)**	**TEN (**μ**g)**
**Reference**		**(62.10** ± **7.17)**	**(9120.78** ± **1097.13)**	**(452.79** ± **102.67)**	**(890.18** ± **516.13)**	**(0.18** ± **0.09)**	**(44.90** ± **18.76)**	**(90.50** ± **9.75)**	**(338.10** ± **152.38)**	**(0.092** ± **0.05)**	**(90.00** ± **0.00)**	**(430.03** ± **32.41)**	**(13.26** ± **0.32)**	**(6.70** ± **2.20)**	**(0.945** ± **0.29)**
TR1	*Streptomyces violaceoruber*	(50.80 ± 3.55)^*^	(4575.50 ± 373.08) ^*^	(456.58 ± 74.11)	(1196.36 ± 403.39)	(0.09 ± 0.00)	(42.25 ± 7.18)	(434.20 ± 48.02)^**^	(1572.63 ± 150.06)^***^	(0.25 ± 0.22)	(90.00 ± 0.00)	(415.39 ± 236.40)	(19.82 ± 168.38)	(24.57 ± 12.58)	(0.61 ± 0.16)
TR3	*Variovorax paradoxus*	(41.60 ± 5.78)^**^	(2013.00 ± 618.26)^**^	(186.05 ± 102.05)^*^	(239.21 ± 86.43)	(0.55 ± 0.52)	(48.83 ± 18.84)	(130.19 ± 49.02)	(271.72 ± 184.40)	(2.08 ± 0.38)	(75.90 ± 10.09)^*^	(411.85 ± 40.58)	(106.37 ± 11.51)^**^	(60.01 ± 26.19)	(9.758 ± 1.56)^**^
TR4	*Rhodococcus qingshengii*	(62.7 ± 5.55)	(3259.16 ± 935.02)^**^	(659.19 ± 102.15)	(1659.65 ± 871.24)	(0.01 ± 0.00)	(33.77 ± 6.05)	(171.68 ± 131.15)	(247.48 ± 191.68)	(1.06 ± 0.37)	(90.00 ± 0.00)	(593.75 ± 107.82)	(10.53 ± 4.71)	(1.39 ± 0.61)^*^	(0.26 ± 0.08)^*^
TR8	*Streptomyces dioscori*	(58.70 ± 5.39)	(6793.66 ± 2604.83)	(790.35 ± 169.97)	(1378.14 ± 289.48)	(0.01 ± 0.00)	(42.00 ± 15.03)	(123.68 ± 62.61)	(436.51 ± 149.21)	(5.60 ± 4.56)	(90.00 ± 0.00)	(625.71 ± 115.95)	(49.94 ± 2.11)^***^	(40.3 ± 12.44)^*^	(0.81 ± 0.12)
TR10	*Leifsonia shinshuensis*	(59.50 ± 3.26)	(4166.31 ± 1781.63)^*^	(481.64 ± 225.56)	(871.70 ± 518.31)	(0.02 ± 0.01)	(43.50 ± 5.84)	(50.56 ± 3.86)^*^	(300.486 ± 76.14)	(23.50 ± 5.41)^*^	(90.00 ± 0.00)	(526.66 ± 139.33)	(49.27 ± 13.67)^*^	(26.26 ± 9.77)	(1.20 ± 0.26)
TR11	*Priestia megaterium*	(59.10 ± 2.70)	(9707.39 ± 1761.41)	(709.73 ± 141.89)^*^	(1720.4 ± 205.09)	(0.03 ± 0.05)	(51.00 ± 3.77)	(86.644 ± 51.31)	(342.10 ± 121.01)	(42.15 ± 15.96)^*^	(90.00 ± 0.00)	(484.55 ± 47.46)	(29.63 ± 5.58)^*^	(28.03 ± 10.73)	(3.18 ± 0.35)^**^
TR13	*Leifsonia xyli*	(53.50 ± 6.27)	(2527.39 ± 173.184)^**^	(178.14 ± 1184.83)^*^	(328.37 ± 127.68)	(0.04 ± 0.06)	(46.60 ± 7.69)	(28.87 ± 10.41)^**^	(137.721 ± 51.65)	(76.55 ± 53.99)	(90.00 ± 0.00)	(486.56 ± 84.08)	(55.58 ± 12.61)^*^	(31.92 ± 10.66)^*^	(0.45 ± 0.09)
TR14	*Microbacterium trichothecenolyticum*	(51.40 ± 2.82)^*^	(5254.52 ± 1600.38)^*^	(285.64 ± 102.56)	(608.35 ± 264.15)	(0.10 ± 0.05)	(48.10 ± 14.44)	(43.68 ± 25.23)	(378.27 ± 121.61)	(16.74 ± 19.40)	(90.00 ± 0.00)	(549.25 ± 91.45)	(26.59 ± 5.63)	(25.23 ± 8.62)	(0.802 ± 0.09)
**TR17**	***Arthrobacter nitroguajacolicus***	(64.10 ± 5.03)	(464.13 ± 298.379)^**^	(461.63 ± 102.00)	(1418.21 ± 427.30)	(0.03 ± 0.02)	(45.90 ± 10.03)	(128.68 ± 70.12)	(408.46 ± 226.92)	(8.63 ± 12.65)	(90.00 ± 0.00)	(8.1714 ± 107.48)^**^	(54.11 ± 31.60)	(23.01 ± 16.43)	(0.09 ± 0.07)^*^
TR18	*Paenibacillus panacihumi*	(49.30 ± 3.25)^*^	(7934.39 ± 952.94)	(817.29 ± 35.20)^*^	(1131.5 ± 275.4)	(0.09 ± 0.01)	(46.80 ± 15.33)	(534.07 ± 379.80)	(2,650.27 ± 3,182.13)	(13.85 ± 20.14)	NG	-	-	-	-
TR27	*Streptomyces clavuligerus*	(58.60 ± 5.95)	(8488.39 ± 2543.21)	(695.00 ± 294.97)	(1211.27 ± 437.99)	(0.06 ± 0.04)	(47.20 ± 16.75)	(117.68 ± 83.06)	(502.62 ± 339.49)	(0.95 ± 0.81)	(90.00 ± 0.00)	(387.82 ± 84.57)	(20.53 ± 12.44)^ns^	(13.89 ± 9.04)	(0.79 ± 0.33)
TR30	*Pseudomonas fluorescens*	(49.40 ± 3.73)^*^	(1700.20 ± 174.63)^**^	(399.74 ± 95.70)	(412.31 ± 156.44)	(0.09 ± 0.10)	(35.62 ± 5.37)	(53.68 ± 20.74)	(180.56 ± 108.07)	(0.74 ± 0.74)	(77.30 ± 3.27)^***^	(283.29 ± 79.98)	(33.47 ± 10.65)	(15.72 ± 7.61)	(3.70 ± 5.03)^*^
TR31	*Chryseobacterium ureilyticum*	(51.70 ± 2.20)^*^	(1993.89 ± 694.74)^**^	(155.70 ± 64.20)^*^	(382.51 ± 200.00)	(0.00 ± 0.04)	(51.5 ± 13.24)	(48.17 ± 5.69)^*^	(365.2 ± 5.82.181)	(120.26 ± 73.84)	(80.00 ± 0.00)	(182.47 ± 12.30)^**^	(58.95 ± 10.29)^*^	(38.47 ± 8.07)^*^	(0.949 ± 0.16)
**TR38**	***Bacillus pumilus***	(47.70 ± 1.60)^**^	(4272.22 ± 780.28)^**^	(645.89 ± 102.7)^*^	(541.42 ± 307.40)	(0.59 ± 0.11)	(40.00 ± 6.50)	(109.55 ± 49.02)	(735.97 ± 184.73)	(57.22 ± 7.08)^**^	(90.00 ± 0.00)	(580.49 ± 113.34)	(580.49 ± 113.34)	(13.80 ± 2.81)^**^	(20.02 ± 1.82)
**TR40**	***Serratia nematodiphila***	(46.20 ± 0.97)^**^	(1368.22 ± 156.99)^**^	(132.11 ± 102.98)^*^	(64.01 ± 7.35)	(0.21 ± 0.06)	(38.40 ± 8.76)	(14.47 ± 17.20)^**^	(51.263 ± 184.13)	(0.08 ± 0.06)^**^	(61.10 ± 0.96)^***^	(81.885 ± 30.3)^***^	(1.53 ± 0.76)^***^	(2.100 ± 0.87)	(0.28 ± 0.17)^*^
TR52	*Microbacterium oleivorans*	(54.10 ± 4.52)	(7618.39 ± 2332.05)	(705.36 ± 197.97)	(1527.11 ± 363.10)	(0.05 ± 0.06)	(39.80 ± 7.99)	(431.68 ± 659.35)	(897.27 ± 1,041.55)	(6.64 ± 4.88)	(90.00 ± 0.00)	(882.28 ± 45.85)^***^	(52.96 ±6.67)^**^	(34.38 ± 12.90)	(1.57 ± 0.73)
TR54	*Chryseobacterium soli*	NG	-	-	-	-	(57.00 ± 11.48)	(450.68 ± 172.12)	(2,291.20 ± 938.59)	(20.23 ± 21.70)	(74.8 ± 1.92)^***^	(410.99 ± 66.87)	(8.45 ± 3.26)	(8.370 ± 8.28)	(0.68 ± 0.24)
TR55	*Stenotrophomonas maltophilia*	(63.80 ± 5.48)	(2831.33 ± 148.68)^**^	(351.29 ± 102.4514)	(340.53 ± 69.27)	(0.07 ± 0.02)	(60.24 ± 13.19)	(436.93 ± 115.42)^*^	(1,196.09 ± 335.44)^*^	(108.00 ± 151.65)	(90.00 ± 0.00)	(589.63 ± 90.63)	(50.85 ± 7.45)^*^	(30.70 ± 10.42)	(0.93 ± 0.09)
TR56	*Pseudomonas thivervalensis*	(50.40 ± 2.19)^*^	(3,008.77 ± 282.21)^**^	(235.54 ± 43.10)	(1,228.16 ± 444.43)	(0.02 ± 0.01)	(35.83 ± 1.58)	(1,904.2 ± 349.02)^*^	(3,569.0 ± 756.90)^*^	(10.03 ± 4.08)	(58.50 ± 3.33)^***^	(619.97 ± 178.17)	(37.47 ± 12.78)	(92.85 ± 62.72)	(0.52 ± 0.49)
**TR57**	***Bacillus safensis***	(44.30 ± 4.88)^**^	(2,093.15 ± 143.55)^**^	(399.79 ± 102.15)	(1,098.03 ± 120.19)	(1.25 ± 0.30)^*^	(26.10 ± 1.38)	(100.57 ± 36.78)	(799.26 ± 136.74)^*^	(32.13 ± 5.99)^*^	(54.00 ± 6.64)^***^	(267.91 ± 28.25)^**^	(36.47 ± 3.93)^**^	(103.29 ± 48.42)	(2.67 ± 1.85)
TR58	*Paenibacillus amylolyticus*	(53.80 ± 2.75)	(8,251.39 ± 661.39)	(1,025.5 ± 5397.2)^*^	(1,865.30 ± 211.02)	(0.05 ± 0.04)	(49.99 ± 16.13)	(393.68 ± 175.61)	(1,194.42 ± 264.28)^**^	(3.89 ± 0.37)	NG	-	-	-	-
**TR59**	***Bacillus pumilus***	(52.90 ± 3.13)^*^	(4,938.13 ± 937.23)^**^	(610.20 ± 221.10)	(947.81 ± 334.83)	(0.06 ± 0.03)	(50.00 ± 10.40)	(29.26 ± 5.04)^**^	(206.855 ± 59.35)	(114.30 ± 127.52)	(74.80 ± 5.64)^**^	(786.10 ± 74.13)^**^	(5.58 ± 0.92)^**^	(6.392 ± 0.47)	(1.73 ± 0.36)^*^
TR60	*Pseudomonas koreensis*	(66.50 ± 9.78)	(583.07 ± 319.90)^**^	(57.565 ± 6,909.90)^*^	(303.06 ± 116.69)	(0.05 ± 0.00)	(32.94 ± 2.78)	(42.24 ± 12.47)^*^	(220.264 ± 34.87)	(0.33 ± 0.26)	(66.30 ± 37.08)	(478.80 ± 129.77)	(74.64 ± 11.84)^*^	(49.51 ± 14.87)^*^	(7.24 ± 1.42)^*^
TR61	*Enterobacter asburiae*	(53.40 ± 1.85)	(3,156.03 ± 228.04)^**^	(363.09 ± 102.068)	(911.19 ± 325.74)	(0.03 ± 0.01)	(44.51 ± 2.71)^*^	(1229.97 ± 78.90)^**^	(2,795.99 ± 323.29)^**^	(9.58 ± 6.58)	(67.40 ± 2.98)^***^	(835.87 ± 9.54)^**^	(15.77 ± 1.14)	(17.54 ± 2.11)^**^	(3.24 0.68)^*^
**TR62**	***Bacillus subtilis***	(20.10 ± 3.11)^***^	(44.52 ± 5.51)^**^	(214.32 ± 102.90)	(88.97 ± 5.80)	(0.00 ± 0.00)	(19.37 ± 1.6.70)	(143.68 ± 47.65)	(197.24 ± 104.03)	(0.17 ± 0.10)	(210 ± 2.17)^***^	(19.659 ± 7.13)^**^	(49.97 ± 26.83)	(346.10 ± 212.60)	(0.01 ± 0.00)^*^
**TR65**	***Variovorax boronicumulans***	(69.70 ± 14.70)	(117.58 ± 26.24)^**^	(269.49 ± 102.577)	(114.67 ± 50.73)	(0.93 ± 0.03)	(55.20 ± 14.86)	(65.854 ± 41.97)	(177.45 ± 184.88)	(1.61 ± 1.42)	(90.00 ± 0.00)	(7.0811 ± 4.18)^**^	(82.82 ± 46.57)	(28.12 ± 9.20)^*^	(1.44 ± 0.52)
TR66	*Streptomyces griseoaurantiacus*	(47.30 ± 2.82)^**^	(4,251.26 ± 1,543.38)^*^	(699.64 ± 214.56)	(1,361.55 ± 440.28)	(0.04 ± 0.03)	(43.07 ± 10.13)	(224.68 ± 193.05)	(756.53 ± 392.76)	(1.69 ± 1.69)	(90.00 ± 0.00)	(657.15 ± 77.18)^*^	(33.73 ± 11.2)	(18.28 ± 11.81)	(1.34 ± 0.38)
TR72	*Stenotrophomonas maltophilia*	(60.75 ± 33.91)	(5,725.39 ± 2,022.74)	(797.82 ± 228.97)	(1,781.23 ± 625.21)	(0.11 ± 0.07)	(62.30 ± 18.91)	(216.30 ± 192.01)	(482.14 ± 366.69)	(1.46 ± 1.13)	(90.00 ± 0.00)	(700.14 ± 29.24)^***^	(78.49 ± 47.50)	(47.80 ± 29.65)	(1.05 ± 0.42)
TR82	*Stenotrophomonas maltophilia*	(75.13 ± 33.60)	(1,882.18 ± 339.29)^**^	(195.69 ± 8,297.85)^*^	(179.45 ± 43.07)	(0.09 ± 0.03)	(69.80 ± 6.054)	(115.68 ± 24.70)	(447.804 ± 155.3)	(10.85 ± 1.70)^**^	(90.00 ± 0.00)	(537.96 ± 52.88)	(111.13 ± 5.77)^**^	(49.33 ± 11.36)^*^	(1.56 ± 0.22)^*^
TR84	*Chitinophaga polysaccharea*	(55.00 ± 3.54)	(2,950.97 ± 785.91)^**^	(412.61 ± 102.245)	(342.90 ± 98.41)	(0.11 ± 0.00)	(49.70 ± 3.31)	(25.98 ± 5.88)^**^	(185.27 ± 127.00)	(60.05 ± 17.16)^*^	(90.00 ± 0.00)	(230.87 ± 50.49)^**^	(12.17 ± 5.06)	(8.32 ± 3.89)	(10.62 ± 1.73)^**^
TR88	*Pseudomonas brassicacearum*	(56.40 ± 2.97)	(4,836.79 ± 216.45)^*^	(312.64 ± 129.56)	(1,991.90 ± 312.2)^*^	(0.13 ± 0.54)	(52.00 ± 3.40)	(236.36 ± 109.91)	(1,788.28 ± 398.58)^*^	(1.01 ± 1.42)	(70.60 ± 2.48)^***^	(819.94 ± 137.97)^*^	(5.08 ± 1.09)^**^	(6.04 ± 2.043)	(0.416 ± 0.37)
TR91	*Luteibacter rhizovicinus*	(46.40 ± 1.85)^**^	(2,589.06 ± 645.85)^**^	(352.55 ± 102.23)	(1331.63 ± 276.13)	(0.37 ± 0.21)	(38.75 ± 0.96)	(206.16 ± 33.74)	(977.79 ± 175.80)^**^	(0.23 ± 0.03)	(36.30 ± 33.1)^**^	(280.10 ± 89.00)	(3.58 ± 2.40)^*^	(2.27 ± 1.48)	(3.61 ± 0.41)^**^
**TR92**	***Bacillus subtilis***	(22.30 ± 1.44)^***^	(28.89 ± 10.16)^**^	(237.44 ± 102.69^*^	(195.17 ± 127.89	(0.04 ± 0.03)	(15.70 ± 2.95)^*^	(107.68 ± 11.98)	(245.27 ± 35.073)	(0.05 ± 0.03)	(21.60 ± 0.96)^***^	(10.12 ± 5.42)^**^	(23.70 ± 10.65)	(205.99 ± 93.48)	(0.01 ± 0.00)^*^
TR93	*Priestia megaterium*	(56.20 ± 7.29)	(4,435.79 ± 304.45)^*^	(522.64 ± 110.56)	(977.56 ± 182.01)	(0.04 ± 0.54)	(54.60 ± 6.08)	(111.70 ± 17.02)	(482.84 ± 184.29)	(80.42 ± 95.53)	(90.00 ± 0.00)	(545.85 ± 109.36)	(19.37 ± 5.38)	(18.58 ± 9.06)	(3.50 ± 0.28)^***^
**TE98**	***Serratia nematodiphila***	(45.40 ± 2.68)^**^	(1,235.80 ± 143.99)^**^	(193.52 ± 102.13)^*^	(90.45 ± 66.21)	(0.12 ± 0.05)	(30.50 ± 4.17)	(4.8766 ± 1.02)	(8.76 ± 184.86)	(0.01 ± 0.00)	(64.10 ± 4.35)^***^	(83.436 ± 12.07)^**^	(1.37 ± 1.03)^**^	(9.791 ± 13.01)	(0.52 ± 0.27)
TE99	*Enterobacter ludwigii*	(45.60 ± 1.47)^**^	(1,755.99 ± 186.71)^**^	(266.66 ± 102.80)	(203.08 ± 63.52)	(0.19 ± 0.07)	(33.00 ± 3.84)	(10.78 ± 8.44)^***^	(24.78 ± 19.60)	(0.07 ± 0.06)	(78.80 ± 6.69)^*^	(99.699 ± 29.64)^***^	(0.8068 ± 0.20)^***^	(1.86 ± 0.58)	(0.55 ± 0.19)
TE103	*Enterobacter asburiae*	(49.10 ± 0.82)^*^	(2,196.51 ± 210.80)^**^	(309.68 ± 14.80)	(150.02 ± 26.55)	(0.20 ± 0.00)	(38.00 ± 2.47)	(11.69 ± 3.11)^**^	(12.93 ± 6.56)	(0.03 ± 0.04)	(80.60 ± 4.37)^**^	(127.44 ± 58.33)^**^	(2.61 ± 0.35)^***^	(3.36 ± 0.44)	(0.99 ± 0.29)
TE105	*Pseudomonas citronellolis*	(48.80 ± 4.66)^*^	(4,131.86 ± 720.82)^**^	(657.42 ± 179.60)	(2,146.91 ± 558.56)^*^	(0.04 ± 0.02)	(47.00 ± 8.4)	(59.68 ± 37.04)	(266.04 ± 186.15)	(66.65 ± 106.63)	NG	-	-	-	-
**TE106**	***Bacillus amyloliquefaciens***	(24.30 ± 4.84)^***^	(34.71 ± 12.01)^**^	(259.63 ± 102.98)	(259.60 ± 73.57)	(0.02 ± 0.01)	(12.70 ± 1.20)^*^	(59.303 ± 24.02)	(163.22 ± 61.79)	(2.83 ± 4.81)	(23.80 ± 0.75)^***^	(15.675 ± 7.71)^**^	(40.37 ± 20.80)	(252.0 ± 120.07)	(0.01 ± 0.01)^*^
**TE108**	***Kosakonia cowanii***	(46.90 ± 4.26)^**^	(2,087.21 ± 242.30)^**^	(314.84 ± 102.87)	(316.59 ± 31.94)	(0.12 ± 0.01)	(43.30 ± 2.05)	(11.12 ± 6.48)^**^	(34.19 ± 28.49)	(4.49 ± 2.88)	(83.50 ± 2.23)^**^	(203.10 ± 43.46)^**^	(1.465.± 2.09)^**^	(3.34 ± 4.73)	(6.10 ± 1.58)^*^
**TE109**	***Pantoea agglomerans***	(48.90 ± 3.49)^*^	(2,002.18 ± 685.95)^**^	(321.29 ± 113.40)	(159.35 ± 28.71)	(0.19 ± 0.05)	(31.90 ± 4.60)	(19.434 ± 7.02)^**^	(42.18 ± 8.77)	(4.70 ± 7.58)	(75.60 ± 5.28)^**^	(82.672 ± 21.05)^***^	(4.37 ± 1.756)^*^	(5.60 ± 2.12)	(0.72 ± 0.45)
**TE110**	***Serratia nematodiphila***	(47.20 ± 1.86)^**^	(1,350.93 ± 311.70)^**^	(242.08 ± 102.67)	(73.34 ± 27.25)	(0.08 ± 0.07)	(33.60 ± 7.97)	(7.4571 ± 7.521)	(8.76 ± 6.26)	(6.14 ± 10.58)	(67.20 ± 5.56)^***^	(153.43 ± 45.03)^**^	(2.37 ± 2.125)^**^	(1.84 ± 0.89)^*^	(0.41 ± 0.13)
TE114	*Pseudomonas nitroreducens*	(46.70 ± 1.60)^**^	(5,834.23 ± 1233.38)^*^	(1,606.64 ± 521.56)	(4,026.8 ± 923.18)^*^	(0.07 ± 0.00)	(47.80 ± 8.12)	(25.41 ± 7.06)^**^	(220.261 ± 90.76)	(216.78 ± 51.77)^*^	(800 ± 35.7)^*^	(620.94 ± 40.23)	(48.3± 5.92)^**^	(24.33 ± 9.28)	(1.39 ± 0.10)
**TE116**	***Pantoea agglomerans***	(47.70 ± 1.04)^*^	(2,216.97 ± 422.16)^**^	(420.57 ± 82.00)	(196.58 ± 78.28)	(0.16 ± 0.06)	(33.70 ± 4.60)	(30.800 ± 5.02)^**^	(61.490 ± 184.47)	(1.78 ± 1.00)	(75.30 ± 5.48)^**^	(116.85 ± 13.85)^**^	(3.37 ± 0.418)^***^	(4.38 ± 0.78)	(1.22 ± 0.42)
**TE117**	***Serratia marcescens***	(47.60 ± 2.27)^**^	(1,618.42 ± 358.03)^**^	(285.94 ± 102.60)	(69.98 ± 40.52)	(0.23 ± 0.09)	(36.80 ± 1.79)	(7.69 ± 4.17)^**^	(14.1454 ± 7.06)	(11.10 ± 11.90)	(57.90 ± 7.82)^***^	(91.12 ± 12.68)^**^	(1.64 ± 0.62)^***^	(4.95 ± 1.92)	(0.83 ± 0.38)

The parameters measured are fungal growth (mm), production of tenuazonic acid (TeA), alternariol (AOH), alternariol monomethyl ether (AME), and tentoxin (TEN). Results are expressed in average ± standard deviation. NG = no growth thus mycotoxins values were not measured. Asterisks indicate the statistical significance between the values measured for the reference and the one for the isolate

* *P* ≤ 0.05,

** *P* ≤ 0.01, and

*** *P* ≤ 0.001.

Among the tested strains, *Bacillus* species, especially *B. amyloliquefaciens* TE106, *B. subtilis* TR92, and TR62, were found to be the most effective in reducing the fungal growth of *A. alternata* by 60–67% (*P* < 0.001) and *A. tenuissima* by 73–76% (*P* < 0.001), and the only isolates able to significantly reduce *A. solani* by 57–71% (*P* < 0.05). Other *Bacillus* isolates, such as *B. pumilus* TR38 and TR59, *B. safensis* TR57, showed moderate reductions in fungal biomass of *A. alternata*, reaching reductions of 44–52% (*P* < 0.01 for TR38 and TR57 and *P* < 0.05 for TR59) and reductions of *A. tenuissima* of 42% (*P* < 0.001) by TR57 and 17% (*P* < 0.01) by TR59. Generally, *Bacillus* species are well-known to be effective biocontrol agents, and research has demonstrated their efficacy several times, performing *in vitro* and *in vivo* experiments, where plant defense was enhanced by their biocontrol activity (Yang et al., 2023). *Bacillus* antagonistic action is often associated with the production of bacteriocins, antimicrobial peptides, and especially cyclic lipopeptides, among which iturin, fengycin, and surfactin are the most effective and most studied (Prakash and Arora, 2021). More recently, the potential of *Bacillus* VOCs as antimicrobial compounds has been studied (Grahovac et al., 2023). Interestingly, the same metabolites that were found to have biological control effects were also found to trigger ISR in plants (Fira et al., 2018; Yang et al., 2023), extending *Bacillus* plant defense potential.

Some other interesting results in fungal biomass reduction were obtained for the isolates *K. cowanii* TE108, *S. nematodiphila* TR40, TE98, TE110, *S. marcescens* TE117, and *P. agglomerans* TE109, TE116. In these cases, reductions varied greatly, according to the *Alternaria* species considered. These genera are all closely related to the *Enterobacter* genus. Indeed, they are all part of the same Enterobacterales order or, in the case of the genera *Enterobacter* and *Kosakonia*, the same *Enterobacteriaceae* family (van Belkum, 2006). Although to a lower extent than *Bacillus* species, these isolates also showed good results in limiting fungal growth, with *A. alternata* inhibition of 24–26% (*P* < 0.01) for *K. cowanii* TE108 and *S. marcescens* TE117, *E. ludwigii* TE99, and 21% (*P* < 0.05) for *E. asburiae* TE103 (Table 1). *A. solani* growth was not affected by these isolates, while *A. tenuissima* was significantly reduced by TE108 (7%, *P* < 0.01), TE117 (35%, *P* < 0.01), TE99 (12%, *P* < 0.05), and TE103 (10%, *P* < 0.01) (Table 1). *Enterobacter* sp. is often found in the rhizosphere (Dong et al., 2019); however, few studies focus on the mode of action by which this genus operates to inhibit fungal growth. Only a few research individuated some potential features related to lytic enzymes, chitinase, and lipase (Xue et al., 2009), while Ghosh and Sarkar (2022) reported a biocontrol effect due to the production of zirconium oxide nanoparticles (ZrONPs) or Herbicolin-A for *P. agglomerans* (Xu et al., 2022). Although promising biocontrol results are obtained by many authors for *Enterobacter, Kosakonia, Pantoea*, and *Serratia* (Guo et al., 2020), and are found in this study, their application as either biostimulants or biocontrol agents may be tricky due to their similarity to human pathogenic *Enterobacteriaceae* from which they most likely inherited some pathogenicity traits. The potential applications of microorganisms should not be disregarded *a priori*, but further research should be conducted with caution. It is important to consider virulence factors and antimicrobial resistance genes and to follow a thorough risk assessment procedure prior to any applications (PPR-EFSA, 2013).

Additionally, some Pseudomonas species successfully reduced fungal biomass; in particular *P. fluorescens* TR30, which inhibited fungal growth of *A. alternata* by 20% (*P* < 0.05) and 14% *A. tenuissima* (*P* < 0.001), *P. thivervalensis* TR56 19% (*P* < 0.05) 35% (*P* < 0.001), and *P. brassicacearum* TR88 active only on *A. tenuissima* with reduction of 22% (*P* < 0.001) (Table 1). The *Pseudomonas* genus is well-established among the BCAs, and their main effects are imputed to the production of antimicrobial compounds such as 2,4-diacetylphloroglucinol (PHL), which plays a major role in biological control (Rezzonico et al., 2007) but also other secondary metabolites such as pyoluteorin, pyrrolnitrin, and phenazines have been proved to be successful and most of them are also implied in ISR other than being active antibiotic molecules (Haas and Keel, 2003).

**Figure 1:**
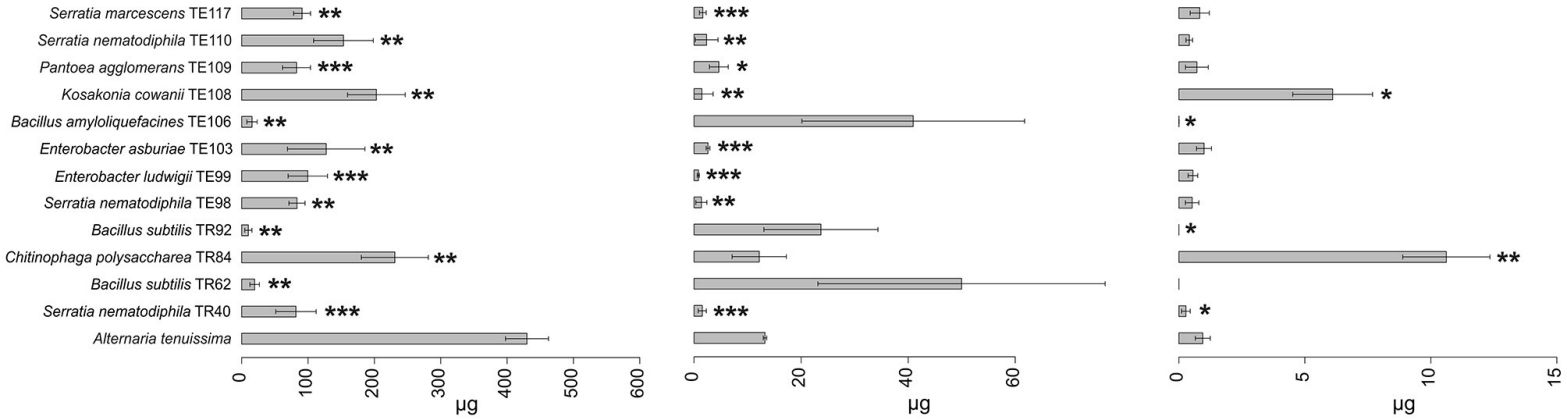
Barplot representing average production (μg ± SD) of tenuazonic acid (TeA), alternariol (AOH), and tentoxin (TEN) mycotoxins by *A. tenuissima* in the presence of the top 12 strains in the ranking and in the absence of any strain (reference bar at the bottom of the barplot). Asterisks indicate the statistical significance between the values measured for the reference and the one for the isolate *P ≤ 0.05, **P ≤ 0.01, and ***P ≤ 0.001. Alternariol monomethyl ether (AME) results were not shown since no statistically significant results were observed in any of the strains.

**Figure 2:**
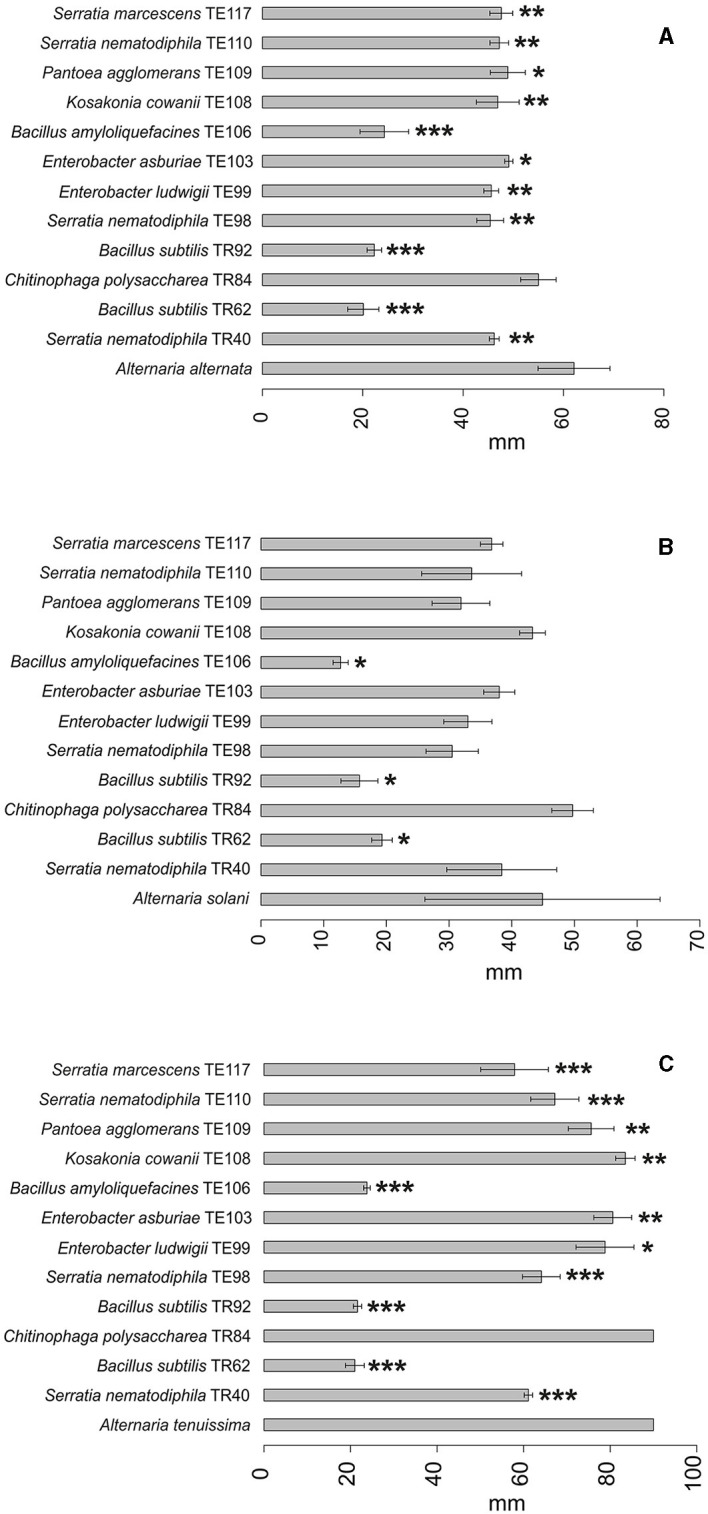
Barplot representing average growth diameter (mm±SD) of *A. alternata* **(A)**, *A. solani* **(B)**, and *A. tenuissima* **(C)** in the presence of the top 12 strains in the ranking and in the absence of any strain (reference bar at the bottom of the barplot). Asterisks indicate the statistical significance between the values measured for the reference and the one for the isolate *P ≤ 0.05; **P ≤ 0.01; and ***P ≤ 0.001.

**Figure 3:**
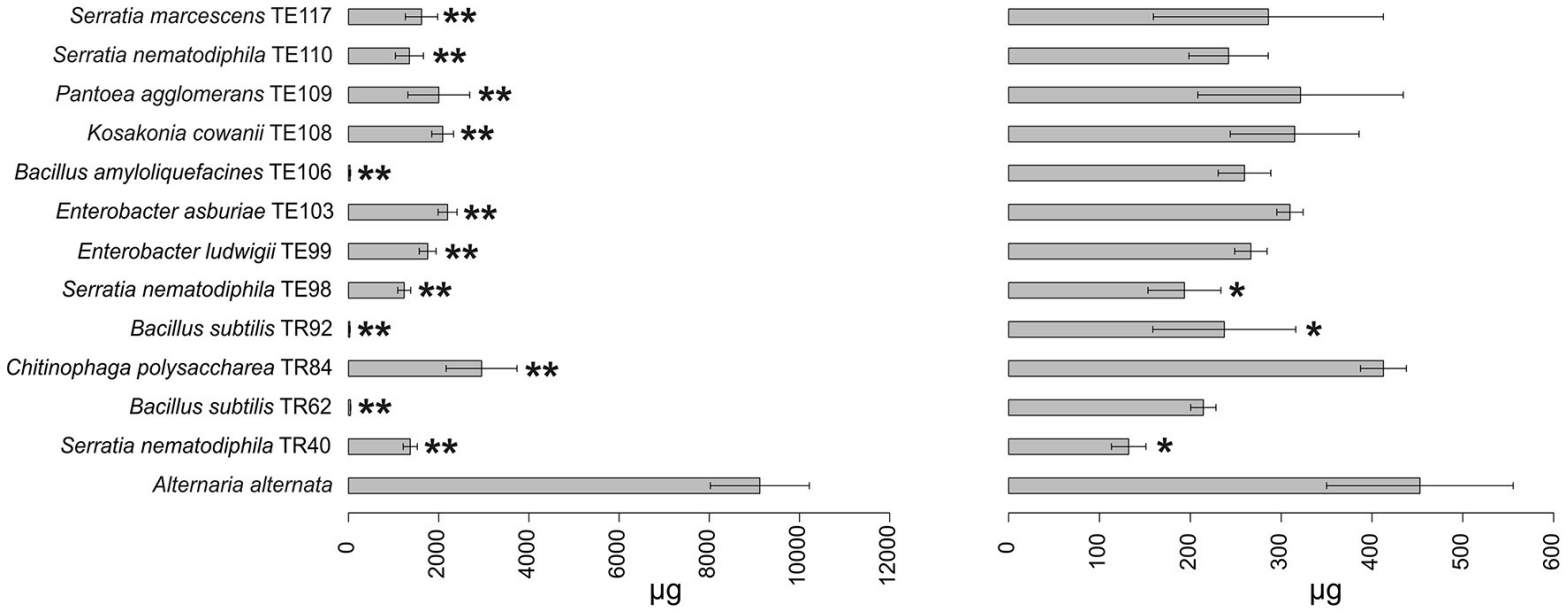
Barplot representing average production (μg ± SD) of tenuazonic acid (T) and alternariol (AOH) mycotoxins by *A. alternata* in the presence of the top 12 strains in the ranking and in the absence of any strain (reference bar at the bottom of the barplot). Asterisks indicate the statistical significance between the values measured for the reference and the one for the isolate *P ≤ 0.05; **P ≤ 0.01; and ***P ≤ 0.001. Alternariol monomethyl ether (AME) and tentoxin (TEN) were not shown since no statistically significant results were observed in any of the strains.

**Figure 4:**
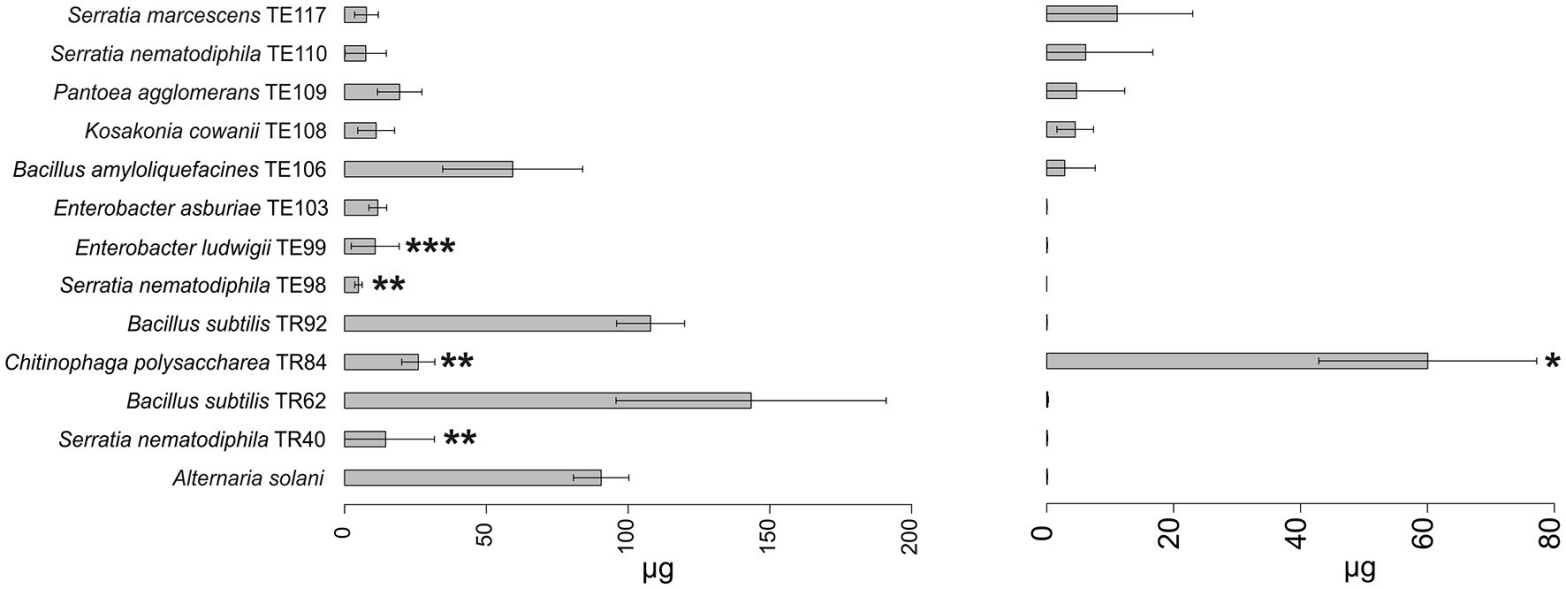
Barplot representing average production (μg ± SD) of alternariol (AOH) and tentoxin (TEN) mycotoxins by *A. solani* in the presence of the top 12 strains in the ranking and in the absence of any strain (reference bar at the bottom of the barplot). Asterisks indicate the statistical significance between the values measured for the reference and the one for the isolate *P ≤ 0.05; **P ≤ 0.01; and ***P ≤ 0.001. Alternariol monomethyl ether (AME) results were not shown since no statistically significant results were observed in any of the strains. Tenuazonic acid (TeA) was not produced by *A. solani*.

Results obtained in mycotoxins reductions were very different, considering both the different bacterial strains and the different *Alternaria* toxins. Among the mycotoxins measured, TEN had a different behavior in comparison with all the other toxins i.e., only few bacterial strains resulted able to reduce it significantly (Figure 1) while in many cases TEN was greatly increased by the interaction with the rhizobacteria (Table 1, Figures 1, 2). Regarding AOH, if we consider reductions higher than 75% with a *p-*value of < 0.05, such results were obtained by *P. koreensis* TR60 (87%) for *A. alternata, E. ludwigii* TE99, *E. asburiae* TE103, *K. cowanii* TE108 (87–88%), *P. agglomerans* TE109 (78%), *S. nematodiphila* TE110, and *S. marcescens* (91–92%) for *A. solani*. *A. tenuissima* production of AOH was controlled by *S. nematodiphila* TR40 and TE98 (88%), by *E. ludwigii* TE99, *E. asburiae* TE103, *K. cowanii* TE108 (94, 80, and 89%, respectively), *S. nematodiphila* TE110, and *S. marcescens* TE117 (82–87%, respectively). Overall, the “*Enterobacter* group” resulted the best in reducing the production of AOH overall, while only TR92 *B. subtilis* was able to reduce AOH by 46% for *A. alternata* only (Table 1 and Figure 3). Interestingly, although not significantly, AOH produced by *A. tenuissima* increased visibly in the assay with the three *Bacillus* species (TR62, TR92, and TE106) with greater impact on its growth indicating a sort of defense response of *A. tenuissma* (Figure 4). AME in *A. alternata* was never reduced significantly due to the high standard deviation measured in the control. The only significant reductions for AME were found for *A. tenuissima* by the strains *Rhodococcus qingshengii* TR4 (80%) and *S. nematodiphila* TE110 (74%) (Figure 4). No significant reductions were obtained for the strains *B. subtilis* TR92, TR62, *Variovorax boronicumulans* TR65, *E. asburiae* TE103, and *E. ludwigii* TE99 (Supplementary Table S3). TeA was never produced by *A. solani* during our experiments, although it was produced by *A. tenuissima* and *A. alternata*. Many strains were able to significantly reduce TeA levels above 75% (Table 1 and Supplementary Table S3), and in almost all cases, if a strain had a good TeA reduction for one *Alternaria* species, the other was equally affected. Twenty bacterial strains reduced the production of this mycotoxin for *A. alternata* by more than 75%, while 12 showed the same result for *A. tenuissima* (Supplementary Table S3). The best reduction effects (*P* < 0.01) were measured for *A. alternata* and *A. tenuissima* by *Arthrobacter nitroguajacolicus* TR17 (95–98%), *B. subtilis* TR62 (99–95%), *V. boronicumulans* TR65 (99–98%), *B. subtilis* TR92 (99–98%), and *B. amyloliquefaciens* TE106 (99–96%) (Table 1).

### 3.3. Ranking system of PGPR antifungal ability and PCA

Considering the complexity of the results, a ranking approach was developed to sort the bacterial strains according to their overall ability to perform antifungal activity. The ranking system used to evaluate the biocontrol ability of each strain was based on the values reported in Supplementary Table S1. The sum of the scores obtained in the ranking for each *Alternaria* species is reported in Table 2 and allowed the individuation of 12 rhizobacteria which resulted in an overall positive score in the global ranking. Among the 12 rhizobacteria individuated, three main bacterial genera dominate: *Bacillus, Serratia*, and *Enterobacter*. *Bacillus* isolates *B. amyloliquefaciens* TE106, *B. subtilis* TR92, and TR62 were shown to be the most capable of decreasing the fungal growth of all *Alternaria* species (Figures 2A–C), with total rankings of 8.50, 11.50, and 5.50, respectively (Table 2). Other *Bacillus* did not rank top in the list due to the limited inhibition of mycotoxin production and poor control of *A. solani* (Table 1). On the other hand, *Serratia* species and *Enterobacter*, as well as *K. cowanii* TE108 and *P. agglomerans* TE109, rank among the top isolates as they all inhibit the fungal growth of *A. alternata* and *A. tenuissma* and performed overall well in the reduction of *Alternaria* toxins inhibiting also AOH production in *A. solani* (Table 1). Finally, *Chitinophaga polysaccharea* TR84, despite being unable to inhibit fungal development, was particularly good at reducing AOH in all *Alternaria* species and also affecting other mycotoxins (Table 1). The barplots provide a visual representation of the differences in fungal growth among the best-performing strains individuated by the ranking system (Figures 1–4).

**Table 2:** Ranking scores assigned to each bacterial strain, based on their ability in reducing *Alternaria* species (*A. solani, A. tenuissima*, and *A. alternata*) growth and their production of mycotoxins: tenuazonic acid (TeA), alternariol (AOH), and alternariol.

**Strain**	**Taxonomy**	***A. alternata***						***A. solani***					***A. tenuissima***						**Global ranking**
		**Fungal growth**	**TeA**	**AOH**	**AME**	**TEN**	**Ranking**	**Fungal growth**	**AOH**	**AME**	**TEN**	**Ranking**	**Fungal growth**	**TeA**	**AOH**	**AME**	**TEN**	**Ranking**	
TR1	*Streptomyces violaceoruber*	0.50	1.50	−1.00	−2.00	1.50	0.50	0.50	−3.00	−3.00	−3.00	−8.50	0.00	0.50	−3.00	−3.00	1.00	−4.50	−12.50
TR3	*Variovorax paradoxus*	1.00	2.00	1.50	1.50	−3.00	3.00	−1.00	−2.00	0.50	−3.00	−5.50	0.50	0.50	−3.00	−3.00	−3.00	−8.00	−10.50
TR4	*Rhodococcus qingshengii*	−1.00	1.50	−2.00	−3.00	2.00	−2.50	0.50	−3.00	1.00	−3.00	−4.50	0.00	−2.00	0.50	2.00	1.50	2.00	−5.00
TR8	*Streptomyces dioscori*	0.50	1.00	−3.00	−3.00	2.00	−2.50	0.50	−2.00	−2.00	−3.00	−6.50	0.00	−2.00	−3.00	−3.00	0.50	−7.50	−16.50
TR10	*Leifsonia shinshuensis*	0.50	1.50	−1.00	0.50	2.00	3.50	0.50	1.00	0.50	−3.00	−1.00	0.00	−1.00	−3.00	−3.00	−2.00	−9.00	−6.50
TR11	*Priestia megaterium*	0.50	−1.00	−3.00	−3.00	1.00	−5.50	−1.00	0.50	−1.00	−3.00	−4.50	0.00	−1.00	−3.00	−3.00	−3.00	−10.00	−20.00
TR13	*Leifsonia xyli*	0.50	1.50	1.50	1.50	1.50	6.50	−1.00	1.50	1.50	−3.00	−1.00	0.00	−1.00	−3.00	−3.00	1.50	−5.50	0.00
TR14	*Microbacterium trichothecenolyticum*	0.50	1.00	1.00	1.00	1.00	4.50	−1.00	1.50	−1.00	−3.00	−3.50	0.00	−2.00	−3.00	−3.00	0.50	−7.50	−6.50
**TR17**	***Arthrobacter nitroguajacolicus***	−1.00	2.00	−1.00	−3.00	2.00	−1.00	−1.00	−2.00	−1.00	−3.00	−7.00	0.00	2.00	−3.00	−3.00	2.00	−2.00	−10.00
TR18	*Paenibacillus panacihumi*	0.50	0.50	−3.00	−2.00	2.00	−2.00	−1.00	−3.00	−3.00	−3.00	−10.00	–	–	–	–	–	–	−12.00
TR27	*Streptomyces clavuligerus*	0.50	0.50	−3.00	−2.00	1.50	−2.50	−1.00	−2.00	−2.00	−3.00	−8.00	0.00	0.50	−3.00	−3.00	0.50	−5.00	−15.50
TR30	*Pseudomonas fluorescens*	0.50	2.00	0.50	1.50	1.00	5.50	1.00	1.00	1.00	−3.00	0.00	0.50	1.00	−3.00	−3.00	−3.00	−7.50	−2.00
TR31	*Chryseobacterium ureilyticum*	0.50	2.00	1.50	1.50	1.50	7.00	−1.00	1.00	−1.00	−3.00	−4.00	0.50	1.50	−3.00	−3.00	0.50	−3.50	−0.50
**TR38**	***Bacillus pumilus***	0.50	1.50	−2.00	1.00	−3.00	−2.00	0.50	−1.00	−3.00	−3.00	−6.50	0.00	−2.00	−1.00	−3.00	−1.00	−7.00	−15.50
**TR40**	***Serratia nematodiphila***	1.00	2.00	1.50	2.00	−3.00	3.50	0.50	2.00	2.00	0.50	5.00	1.00	2.00	2.00	1.50	1.50	8.00	16.50
TR52	*Microbacterium oleivorans*	0.50	0.50	−3.00	−3.00	1.50	−3.50	0.50	−3.00	−3.00	−3.00	−8.50	0.00	−3.00	−3.00	−3.00	−3.00	−12.00	−24.00
TR54	*Chryseobacterium soli*	–	–	–	–	–	–	−1.00	−3.00	−3.00	−3.00	−10.00	0.50	0.50	1.00	−1.00	1.00	2.00	−8.00
TR55	*Stenotrophomonas maltophilia*	−1.00	1.50	0.50	1.50	1.50	4.00	−2.00	−3.00	−3.00	−3.00	−11.00	0.00	−2.00	−3.00	−3.00	0.50	−7.50	−14.50
TR56	*Pseudomonas thivervalensis*	0.50	1.50	1.00	−2.00	2.00	3.00	0.50	−3.00	−3.00	−3.00	−8.50	1.00	−2.00	−3.00	−3.00	1.00	−6.00	−11.50
**TR57**	***Bacillus safensis***	1.00	2.00	0.50	−1.00	−3.00	−0.50	1.00	−3.00	−3.00	−3.00	−8.00	1.00	1.00	−3.00	−3.00	−3.00	−7.00	−15.50
TR58	*Paenibacillus amylolyticus*	0.50	0.50	−3.00	−3.00	1.50	−3.50	−1.00	−3.00	−3.00	−3.00	−10.00	–	–	–	–	–	–	−13.50
**TR59**	***Bacillus pumilus***	0.50	1.00	−2.00	−1.00	1.50	0.00	−1.00	1.50	1.00	−3.00	−1.50	0.50	−3.00	1.50	0.50	−3.00	−3.50	−5.00
TR60	*Pseudomonas koreensis*	−1.00	2.00	2.00	1.50	2.00	6.50	1.00	1.50	1.00	−3.00	0.50	1.00	−1.00	−3.00	−3.00	−3.00	−9.00	−2.00
TR61	*Enterobacter asburiae*	0.50	1.50	0.50	−1.00	2.00	3.50	0.50	−3.00	−3.00	−3.00	−8.50	1.00	−3.00	−1.00	−3.00	−3.00	−9.00	−14.00
**TR62**	***Bacillus subtilis***	1.50	2.00	1.50	2.00	2.00	9.00	1.50	−3.00	1.00	−3.00	−3.50	2.00	2.00	−3.00	−3.00	2.00	0.00	5.50
**TR65**	***Variovorax boronicumulans***	−1.00	2.00	1.00	2.00	−3.00	1.00	−1.00	1.00	1.00	−3.00	−2.00	0.00	2.00	−3.00	−3.00	−3.00	−7.00	−8.00
TR66	*Streptomyces griseoaurantiacus*	0.50	1.50	−3.00	−3.00	2.00	−2.00	0.50	−3.00	−3.00	−3.00	−8.50	0.00	−3.00	−3.00	−3.00	−2.00	−11.00	−21.50
TR72	*Stenotrophomonas maltophilia*	0.50	1.00	−3.00	−3.00	1.00	−3.50	−2.00	−3.00	−2.00	−3.00	−10.00	0.00	−3.00	−3.00	−3.00	−1.00	−10.00	−23.50
TR82	*Stenotrophomonas maltophilia*	−1.00	2.00	1.50	2.00	1.00	5.50	−3.00	−2.00	−2.00	−3.00	−10.00	0.00	−2.00	−3.00	−3.00	−3.00	−11.00	−15.50
TR84	*Chitinophaga polysaccharea*	0.50	1.50	0.50	1.50	1.00	5.00	−1.00	1.50	1.00	−3.00	−1.50	0.00	1.00	0.50	−1.00	−3.00	−2.50	1.00
TR88	*Pseudomonas brassicacearum*	0.50	1.00	1.00	−3.00	1.00	0.50	−1.00	−3.00	−3.00	−3.00	−10.00	0.50	−3.00	1.50	0.50	1.50	1.00	−8.50
TR91	*Luteibacter rhizovicinus*	1.00	1.50	0.50	−2.00	−3.00	−2.00	0.50	−3.00	−3.00	−3.00	−8.50	1.50	1.00	1.50	1.50	−3.00	2.50	−8.00
**TR92**	***Bacillus subtilis***	1.50	2.00	1.00	3.00	1.50	9.00	1.50	−1.00	1.00	1.00	2.50	2.00	2.00	−3.00	−3.00	2.00	0.00	11.50
TR93	*Priestia megaterium*	0.50	1.50	−1.00	−1.00	2.00	2.00	−1.00	−1.00	−2.00	−3.00	−7.00	0.00	−2.00	−2.00	−3.00	−3.00	−10.00	−15.00
**TE98**	***Serratia nematodiphila***	1.00	2.00	1.50	2.00	1.00	7.50	1.00	2.00	2.00	2.00	7.00	1.00	2.00	2.00	−2.00	1.00	4.00	18.50
TE99	*Enterobacter ludwigii*	1.00	2.00	1.00	2.00	−1.00	5.00	1.00	2.00	2.00	1.00	6.00	0.50	2.00	2.00	1.50	1.00	7.00	18.00
TE103	*Enterobacter asburiae*	0.50	2.00	1.00	2.00	−2.00	3.50	0.50	2.00	2.00	1.50	6.00	0.50	1.50	2.00	1.50	−1.00	4.50	14.00
TE105	*Pseudomonas citronellolis*	0.50	1.50	−2.00	−3.00	1.50	−1.50	−1.00	1.00	0.50	−3.00	−2.50	–	–	–	–	–	–	−4.00
**TE106**	***Bacillus amyloliquefaciens***	1.50	2.00	1.00	1.50	2.00	8.00	1.50	1.00	1.50	−3.00	1.00	1.50	2.00	−3.00	−3.00	2.00	−0.50	8.50
**TE108**	***Kosakonia cowanii***	0.50	2.00	1.00	1.50	1.00	6.00	0.50	2.00	2.00	−3.00	1.50	0.50	1.50	2.00	1.50	−3.00	2.50	10.00
**TE109**	***Pantoea agglomerans***	0.50	2.00	1.00	2.00	−1.00	4.50	1.00	2.00	2.00	−3.00	2.00	0.50	2.00	1.50	0.50	0.50	5.00	11.50
**TE110**	***Serratia nematodiphila***	0.50	2.00	1.00	2.00	1.50	7.00	1.00	2.00	2.00	−3.00	2.00	1.00	1.50	2.00	1.50	1.50	7.50	16.50
TE114	*Pseudomonas nitroreducens*	0.50	1.00	−3.00	−3.00	1.50	−3.00	−1.00	1.50	1.00	−3.00	−1.50	0.50	−2.00	−3.00	−3.00	−2.00	−9.50	−14.00
**TE116**	***Pantoea agglomerans***	0.50	2.00	0.50	2.00	1.00	6.00	0.50	1.50	2.00	−3.00	1.00	0.50	1.50	1.50	1.00	−2.00	2.50	9.50
**TE117**	***Serratia marcescens***	0.50	2.00	1.00	2.00	−1.00	4.50	0.50	2.00	2.00	−3.00	1.50	1.00	2.00	2.00	1.00	0.50	6.50	12.50

**Figure 5:**
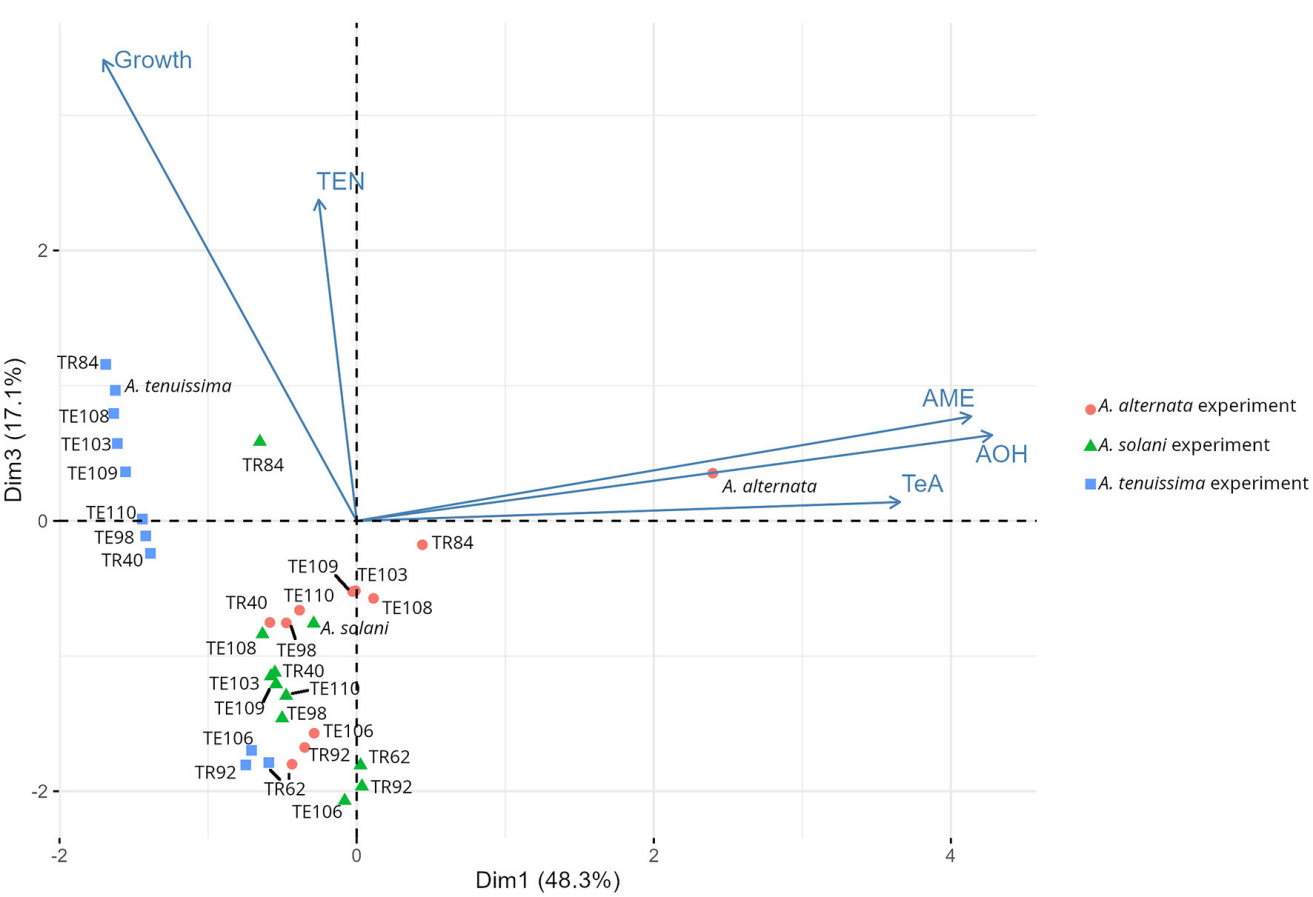
Principal component analysis (PCA) among isolates on the fungal growth diameter (Growth) and on the production of fungal mycotoxins tenuazonic acid (TeA), alternariol (AOH), alternariol monomethyl ether (AME), and tentoxin (TEN). Average points are represented (*n* = 5 for growth, *n* = 3 for mycotoxins). Data correspond to those collected in the experiments with *A. alternata* (red circles), *A. solani* (green triangles), and *A. tenuissima* (blue squares).

Principal Component Analysis (PCA) obtained individually from datasets related to each *Alternaria* species (Supplementary Figures S1–S3) confirms the results obtained from the ranking, since visibly the three *Bacillus* strains TR62, TR92, and TE106 cluster separately in each of the three plots, thus making the ranking system adopted a reliable method for the screening and selecting useful BCAs. The subsequent PCA model prepared considering all the isolates (only the ones highlighted by the ranking are shown) and divided by each *Alternaria* species is reported in Figure 5. The two dimensions selected for the model explain 48.3 and 17.1% of the total variance among samples, respectively. The model shows how *Alternaria* species behaved differently: *A. alternata* was impacted mainly for its mycotoxin production, while *A. tenuissima* for fungal growth. Indeed, *A. tenuissma* growth was altered only by the three *Bacillus* TR62, TR92, and TE106, confirming what is reported in Table 1 and visible in Figure 2C.

Considering the variability in the data obtained from the different *Alternaria* species for each strain tested, and given that several distinct pathogens are expected to co-exist on a single plant, the development of a consortium may lead to better results in regard to plant protection, with a broader spectrum of action and less impacted by environmental variabilities to which a field is normally subjected throughout the crop season (Singh et al., 2023). Although several of the mentioned studies demonstrated some biocontrol activity of PGPR during *in vitro* tests or *in vivo* experiments, the mechanisms by which interaction occurs between PGPR and phytopathogenic fungi are not thoroughly addressed. For example, the specific genetic and biochemical pathways involved are not fully understood. Only some studies focused on the mechanisms of action using gene knockouts to report different effectiveness of BCAs (Weng et al., 2013). Knowledge of the mechanisms of action of the microbial control agents is key to guaranteeing their long-term efficacy in the crop environment. In fact, as resistance builds up over time, it is more likely to occur when a single molecule or a single mode of action is applied. Selective pressure hardly operates when multiple mechanisms of biocontrol are ongoing (Cagliari et al., 2019). Furthermore, pot and field studies are necessary to determine the success of potential microbial agents of biocontrol under different environmental conditions, with different plant genotypes, different soil types, and at different times and ratios of application (Collinge et al., 2022; Lahlali et al., 2022).

### 3.4. Plant growth-promoting traits

The biostimulant ability of the rhizobacteria selected after the preliminary screening was determined by carrying out *in vitro* phenotypic tests. Results reported in Table 3 indicate that the majority of the rhizobacteria isolated showing biocontrol activity (determined in the first screening) are also equipped with PGP traits. All of them tested positive for at least one of the plant growth-promoting activities (PGPAs) *in vitro* (Table 3). This confirms the fact that PGPR may harbor both biostimulant and biocontrol activities by suppressing plant pathogens as well as herbivore insects (Pereira et al., 2021) and potentially reducing mycotoxins in the final product. The fact that PGPR can have both biostimulant and biocontrol activities raises questions about how they should be regulated. The potential conflict between these different regulations has led to discussions about the need for a specific regulatory framework that takes into account the dual function of PGPR. However, to date, no such framework exists, and the regulation of PGPR remains subject to the different regulations for biostimulants and biocontrol agents.

**Table 3:** Characterization of plant growth-promoting traits of PGPR showing antifungal properties in the preliminary screening.

**CODE**	**Taxonomy**	**N-fix (Level)**	**P-sol (broth) (%)**	**PSU (%)**	**IAA (μg/mL)**
TR1	*Streptomyces violaceoruber*	2	0.00	0.00	17.01
TR3	*Variovorax paradoxus*	4	19.93	0.00	3.97
TR4	*Rhodococcus qingshengii*	4	9.30	1.73	0.00
TR8	*Streptomyces dioscori*	1	2.83	6.31	17.88
TR10	*Leifsonia shinshuensis*	3	0.86	0.00	0.00
TR11	*Priestia megaterium*	5	63.10	0.00	12.20
TR13	*Leifsonia xyli*	3	0.00	0.00	1.13
TR14	*Microbacterium trichothecenolyticum*	1	11.79	0.00	2.86
**TR17**	***Arthrobacter nitroguajacolicus***	3	7.86	0.00	2.58
TR18	*Paenibacillus panacihumi*	1	1.10	0.00	3.20
TR27	*Streptomyces clavuligerus*	3	8.48	0.00	3.87
TR30	*Pseudomonas fluorescens*	5	19.02	0.00	2.91
TR31	*Chryseobacterium ureilyticum*	1	0.00	16.73	0.75
**TR38**	***Bacillus pumilus***	4	0.67	0.00	0.00
**TR40**	***Serratia nematodiphila***	5	19.89	68.55	2.29
TR52	*Microbacterium oleivorans*	3	4.02	1.01	10.71
TR54	*Chryseobacterium soli*	1	0.62	57.14	6.47
TR55	*Stenotrophomonas maltophilia*	5	0.00	0.00	2.62
TR56	*Pseudomonas thivervalensis*	3	23.57	6.90	2.19
**TR57**	***Bacillus safensis***	4	0.00	48.96	0.00
TR58	*Paenibacillus amylolyticus*	1	4.12	10.36	1.04
**TR59**	***Bacillus pumilus***	4	1.96	0.00	0.00
TR60	*Pseudomonas koreensis*	5	22.04	7.51	11.48
TR61	*Enterobacter asburiae*	4	44.75	0.00	11.66
**TR62**	***Bacillus subtilis***	2	21.51	35.75	2.00
**TR65**	***Variovorax boronicumulans***	3	2.30	0.00	2.05
TR66	*Streptomyces griseoaurantiacus*	3	1.39	0.00	0.00
TR72	*Stenotrophomonas maltophilia*	2	23.36	ND	8.26
TR82	*Stenotrophomonas maltophilia*	3	33.63	0.00	6.35
TR84	*Chitinophaga polysaccharea*	1	13.75	0.00	9.33
TR88	*Pseudomonas brassicacearum*	4	29.42	5.90	10.56
TR91	*Luteibacter rhizovicinus*	5	0.00	15.20	1.23
**TR92**	***Bacillus subtilis***	2	14.76	52.31	0.99
TR93	*Priestia megaterium*	3	31.77	0.00	0.00
**TE98**	***Serratia nematodiphila***	3	40.96	48.27	11.95
TE99	*Enterobacter ludwigii*	4	41.46	40.44	14.33
TE103	*Enterobacter asburiae*	4	28.08	33.33	22.14
TE105	*Pseudomonas citronellolis*	4	4.46	11.37	0.94
**TE106**	***Bacillus amyloliquefaciens***	5	10.92	23.62	2.62
**TE108**	***Kosakonia cowanii***	4	21.28	0.00	9.14
**TE109**	***Pantoea agglomerans***	4	40.42	26.96	15.55
**TE110**	***Serratia nematodiphila***	3	40.31	41.24	11.10
TE114	*Pseudomonas nitroreducens*	3	28.65	12.68	1.08
**TE116**	***Pantoea agglomerans***	3	39.83	23.21	16.21
**TE117**	***Serratia marcescens***	4	38.12	44.82	12.05

Phenotypic results are expressed quantitatively for phosphorus solubilization (P-sol), percent siderophore unit (PSU%), and indole acetic acid (IAA) production while qualitatively for nitrogen fixation (N-fix), according to the halo formed on plates.

## 4. Conclusion

In the development of biocontrol-based plant protection products, PGPR with antagonistic activity toward plant pathogenic fungi are valuable candidates. Among the multitude of isolates studied, many rhizobacteria reduced fungal biomass (−76%) and/or mycotoxins (−99.7%). The ranking system developed highlighted the 12 best-performing among the initial 85 strains. In particular, *B. amyloliquefaciens* and two strains of *B. subtilis* showed the highest efficacy in reducing fungal biomass and mycotoxin production, while isolates such as E. ludwigii, E. asburiae, S. nematodiphila, P. agglomerans, and K. cowanii showed moderate efficacy. Although the results of this study provide valuable insight into the antifungal activity of rhizobacteria against *Alternaria* and their mycotoxin production, further research is necessary to better understand the mechanisms of action and optimize rhizobacteria use for biocontrol. Additionally, the same isolates displayed PGP traits. Bacillus isolates exhibited nitrogen fixation and siderophore production, while other *Enterobacteriaceae* isolates performed well for nitrogen fixation, phosphate solubilization, and IAA production. These findings support the multifaceted properties of PGPRs beneficial for plant nutrition and defense.

Finally, our results suggest that formulated products containing consortia of rhizobacteria could improve the reliability of biocontrol products. The potential of artificial PGPR consortia can be visualized in the PCA plot, where *B. amyloliquefaciens* and two *B. subtilis* strains form a separate cluster, indicating reduced fungal biomass and mycotoxin production. Future research will validate consortium effects using in-planta assays. In conclusion, the ongoing discussion highlights the need for a specific regulatory framework that considers the dual function of PGPR and could facilitate its integration into sustainable agriculture practices.

## Data availability statement

The datasets presented in this study can be found in online repositories. The names of the repository/repositories and accession number(s) can be found below: GenBank, OQ990921-OQ991008.

## Author contributions

GBe contributed to conceptualization, methodology, investigation, validation, formal analysis, visualization, and writing—original draft. MG contributed to conceptualization, methodology, validation, formal analysis, and writing—original draft. PG contributed to conceptualization, methodology, formal analysis, investigation, and writing—original draft. GBu contributed to conceptualization, formal analysis, and methodology. TB contributed to the conceptualization, formal analysis, writing, reviewing, and editing of the manuscript. MA contributed to data elaboration and visualization. EP contributed to fund acquisition, conceptualization, validation, and editing—original draft. All authors contributed to the manuscript and approved the submitted version.

## Footnotes

^1^FAOSTAT (2022). Available online at: https://www.fao.org/faostat/en/#data/QCL/visualize.

## References

[B1] Aichinger G. Del Favero G. Warth B. Marko D. (2021). Alternaria toxins—Still emerging? Compr. Rev. Food Sci. Food Saf. 20, 4390–4406. 10.1111/1541-4337.12803 34323368

[B2] Ambrosini A. Passaglia L. M. P. (2017). Plant growth–promoting bacteria (PGPB): isolation and screening of PGP activities. Curr. Prot. Plant Biol. 2, 190–209. 10.1002/pb.20054 31725969

[B3] Anith K. N. Nysanth N. S. Natarajan C. (2021). Novel and rapid agar plate methods for in vitro assessment of bacterial biocontrol isolates' antagonism against multiple fungal phytopathogens. Lett. Appl. Microbiol. 73, 229–236. 10.1111/lam.13495 33948964

[B4] Barillot C. D. C. Sarde C.-O. Bert V. Tarnaud E. Cochet N. (2013). A standardized method for the sampling of rhizosphere and rhizoplan soil bacteria associated to a herbaceous root system. Ann. Microbiol. 63, 471–476. 10.1007/s13213-012-0491-y

[B5] Bertuzzi T. Rastelli S. Pietri A. Giorni P. (2021). Alternaria toxins in tomato products in Northern Italy in the period 2017-2019. Food Add. Contam. Part B 14, 170–176. 10.1080/19393210.2021.1895325 34078242

[B6] Cagliari D. Dias N. P. Galdeano D. M. Dos Santos E. Á. Smagghe G. Zotti M. J. (2019). Management of pest insects and plant diseases by non-transformative RNAi. Front. Plant Sci. 10, 1319. 10.3389/fpls.2019.01319 31708946 PMC6823229

[B7] Ciampitti M. Cavagna B. (2014). Developments in integrated pest management in Italy. Phytopathol. Mediterr. 53, 379–384. 10.14601/Phytopathol_Mediterr-1342837081627

[B8] Collinge D. B. Jensen D. F. Rabiey M. Sarrocco S. Shaw M. W. Shaw R. H. (2022). Biological control of plant diseases–What has been achieved and what is the direction? Plant Pathol. 71, 1024–1047. 10.1111/ppa.13555

[B9] Di Cello F. Fani R. (1996). A molecular strategy for the study of natural bacterial communities by PCR-based techniques. Minerva Biotecnol. 8, 126–134.

[B10] Dimkpa C. (2016). Microbial siderophores: Production, detection and application in agriculture and environment. Endocyt. Cell Res. 27, 7–16.

[B11] Dong C.-J. Wang L.-L. Li Q. Shang Q.-M. (2019). Bacterial communities in the rhizosphere, phyllosphere and endosphere of tomato plants. PLoS ONE 14, e0223847. 10.1371/journal.pone.0223847 31703074 PMC6839845

[B12] EFSA Arcella D. Eskola M. Gómez Ruiz J. A. (2016). Dietary exposure assessment to Alternaria toxins in the European population. EFSA J. 14, e04654. 10.2903/j.efsa.2016.465436079840

[B13] Fira D. Dimkić I. Beri,ć T. Lozo J. Stanković S. (2018). Biological control of plant pathogens by Bacillus species. J. Biotechnol. 285, 44–55. 10.1016/j.jbiotec.2018.07.044 30172784

[B14] Garganese F. Ippolito A. di Rienzo V. Lotti C. Montemurro C. Sanzani S. M. (2018). A new high-resolution melting assay for genotyping Alternaria species causing citrus brown spot. J. Sci. Food Agric. 98, 4578–4583. 10.1002/jsfa.8986 29505116

[B15] Ghosh S. Sarkar B. (2022). “Biogenic nanoparticles as novel biocontrol agents,” in Microbial Biocontrol: Sustainable Agriculture and Phytopathogen Management (Springer) 301–322. 10.1007/978-3-030-87512-1_13

[B16] Glick B. R. (2012). Plant growth-promoting bacteria: mechanisms and applications. Scientifica 2012, 963401. 10.6064/2012/963401 24278762 PMC3820493

[B17] Grahovac J. Pajčin I. Vlajkov V. (2023). Bacillus VOCs in the context of biological control. Antibiotics 12, 581. 10.3390/antibiotics12030581 36978448 PMC10044676

[B18] Guerrieri M. C. Fanfoni E. Fiorini A. Trevisan M. Puglisi E. (2020). Isolation and screening of extracellular PGPR from the rhizosphere of tomato plants after long-term reduced tillage and cover crops. Plants 9, 668. 10.3390/plants9050668 32466288 PMC7285081

[B19] Guo D.-J. Singh R. K. Singh P. Li D.-P. Sharma A. Xing Y.-X.. (2020). Complete genome sequence of Enterobacter roggenkampii ED5, a nitrogen fixing plant growth promoting endophytic bacterium with biocontrol and stress tolerance properties, isolated from sugarcane root. Front. Microbiol. 11, 580081. 10.3389/fmicb.2020.580081 33072048 PMC7536287

[B20] Haas D. Keel C. (2003). Regulation of antibiotic production in root-colonizing Pseudomonas spp. and relevance for biological control of plant disease. Ann. Rev. Phytopathol. 41, 117–153. 10.1146/annurev.phyto.41.052002.095656 12730389

[B21] Harikrishnan H. Shanmugaiah V. Balasubramanian N. (2014). Optimization for production of Indole acetic acid (IAA) by plant growth promoting Streptomyces sp VSMGT1014 isolated from rice rhizosphere. Int. J. Curr. Microbiol. Appl. Sci. 3, 158–171.

[B22] Heras J. Domínguez C. Mata E. Pascual V. Lozano C. Torres C.. (2015). GelJ–a tool for analyzing DNA fingerprint gel images. BMC Bioinform. 16, 1–8. 10.1186/s12859-015-0703-0 26307353 PMC4549892

[B23] Iotti M. Bonazzi G. (2018). Analysis of the risk of bankruptcy of tomato processing companies operating in the inter-regional interprofessional organization “OI Pomodoro da Industria Nord Italia.” *Sustainability* 10, 947. 10.3390/su10040947

[B24] Jia Q. Fan Y. Duan S. Qin Q. Ding Y. Yang M.. (2023). Effects of Bacillus amyloliquefaciens XJ-BV2007 on growth of alternaria alternata and production of tenuazonic acid. Toxins 15, 53. 10.3390/toxins15010053 36668873 PMC9867350

[B25] Karthika S. Varghese S. Jisha M. S. (2020). Exploring the efficacy of antagonistic rhizobacteria as native biocontrol agents against tomato plant diseases. 3 Biotech 10, 1–17. 10.1007/s13205-020-02306-1 32656053 PMC7320969

[B26] Lahlali R. Ezrari S. Radouane N. Kenfaoui J. Esmaeel Q. El Hamss H.. (2022). Biological control of plant pathogens: A global perspective. Microorganisms 10, 596. 10.3390/microorganisms10030596 35336171 PMC8951280

[B27] Legein M. Smets W. Vandenheuvel D. Eilers T. Muyshondt B. Prinsen E.. (2020). Modes of action of microbial biocontrol in the phyllosphere. Front. Microbiol. 11, 1619. 10.3389/fmicb.2020.01619 32760378 PMC7372246

[B28] Lin H. Jia B. Wu A. (2023). Cytotoxicities of Co-occurring alternariol, alternariol monomethyl ether and tenuazonic acid on human gastric epithelial cells. Food Chem. Toxicol. 171, 113524. 10.1016/j.fct.2022.113524 36442738

[B29] Mehta S. Nautiyal C. S. (2001). An efficient method for qualitative screening of phosphate-solubilizing bacteria. Curr. Microbiol. 43, 51–56. 10.1007/s002840010259 11375664

[B30] Moss M. O. (1984). Conditions and factors influencing mycotoxin formation in the field and during the storage of food. Chemistry and Industry (UK).

[B31] Panebianco S. Lombardo M. F. Anzalone A. Musumarra A. Pellegriti M. G. Catara V.. (2022). Epiphytic and endophytic microorganisms associated to different cultivar of tomato fruits in greenhouse environment and characterization of beneficial bacterial strains for the control of post-harvest tomato pathogens. Int. J. Food Microbiol. 379, 109861. 10.1016/j.ijfoodmicro.2022.109861 35930961

[B32] Parvin I. Mondal C. Sultana S. Sultana N. Aminuzzaman F. M. (2021). Pathological survey on early leaf blight of tomato and in vitro effect of culture media, temperature and pH on growth and sporulation of alternaria solani. Open Access Libr. J. 8, 1–17. 10.4236/oalib.1107219

[B33] Pereira R. V. Filgueiras C. C. Dória J. Peñaflor M. F. G. V. Willett D. S. (2021). The effects of biostimulants on induced plant defense. Front. Agron. 3, 630596. 10.3389/fagro.2021.630596

[B34] Pieterse C. M. J. Zamioudis C. Berendsen R. L. Weller D. M. Van Wees S. C. M. Bakker P. A. H. M. (2014). Induced systemic resistance by beneficial microbes. Ann. Rev. Phytopathol. 52, 347–375. 10.1146/annurev-phyto-082712-102340 24906124

[B35] PPR-EFSA (2013). Guidance on tiered risk assessment for plant protection products for aquatic organisms in edge-of-field surface waters. EFSA J. 11, 3290. 10.2903/j.efsa.2013.3290

[B36] Prakash J. Arora N. K. (2021). Novel metabolites from Bacillus safensis and their antifungal property against Alternaria alternata. Antonie Van Leeuwenhoek 114, 1245–1258. 10.1007/s10482-021-01598-4 34076810

[B37] Publications Office of the European Union. (2022). Commission Recommendation (EU) 2022/553 of 5 April 2022 on monitoring the presence of Alternaria toxins in food, C/2022/2020. Copy; European Union. Available online at: https://op.europa.eu/en/publication-detail/-/publication/1bd43c13-b544-11ec-b6f4-01aa75ed71a1/language-en (accessed July 26, 2023).

[B38] Ray S. Mondal S. Chowdhury S. Kundu S. (2015). Differential responses of resistant and susceptible tomato varieties to inoculation with Alternaria solani. Physiol. Molec. Plant Pathol. 90, 78–88. 10.1016/j.pmpp.2015.04.002

[B39] Rezzonico F. Zala M. Keel C. Duffy B. Moënne-Loccoz Y. Défago G. (2007). Is the ability of biocontrol fluorescent pseudomonads to produce the antifungal metabolite 2, 4-diacetylphloroglucinol really synonymous with higher plant protection? New Phytol. 173, 861–872. 10.1111/j.1469-8137.2006.01955.x 17286834

[B40] Sadowsky M. J. Hur H.-G. (1998). “Use of endogenous repeated sequences to fingerprint bacterial genomic DNA,” in Bacterial Genomes: Physical Structure and Analysis (Boston, MA: Springer US) 399–413. 10.1007/978-1-4615-6369-3_32

[B41] Sanzani S. M. Gallone T. Garganese F. Caruso A. G. Amenduni M. Ippolito A. (2019). Contamination of fresh and dried tomato by Alternaria toxins in southern Italy. Food Add. Contam. A 36, 789–799. 10.1080/19440049.2019.1588998 30943118

[B42] Schwyn B. Neilands J. B. (1987). Universal chemical assay for the detection and determination of siderophores. Analyt. Biochem. 160, 47–56. 10.1016/0003-2697(87)90612-9 2952030

[B43] Shoaib A. Akhtar S. Akhtar N. (2015). Copper tolerance, protein and catalytic activity in phytopathogenic fungus Alternaria alternata. Global NEST J. 17, 664–672. 10.30955/gnj.001513

[B44] Singh A. Yadav V. K. Chundawat R. S. Soltane R. Awwad N. S. Ibrahium H. A.. (2023). Enhancing plant growth promoting rhizobacterial activities through consortium exposure: A review. Front. Bioeng. Biotechnol. 11, 1099999. 10.3389/fbioe.2023.1099999 36865031 PMC9972119

[B45] Tamm L. Thuerig B. Apostolov S. Blogg H. Borgo E. Corneo P. E.. (2022). Use of copper-based fungicides in organic agriculture in twelve European countries. Agronomy 12, 673. 10.3390/agronomy12030673

[B46] Tang A. Haruna A. S. Ab Majid N. M. Jalloh M. B. (2020). Potential PGPR properties of cellulolytic, nitrogen-fixing, phosphate-solubilizing bacteria in rehabilitated tropical forest soil. Microorganisms. 8, 442. 10.3390/microorganisms8030442 32245141 PMC7143980

[B47] van Belkum A. (2006). Bergey's Manual of Systematic Bacteriology (Volume 2, Parts A–C,). Oxford, UK: Blackwell Publishing Ltd.

[B48] Weng J. Wang Y. Li J. Shen Q. Zhang R. (2013). Enhanced root colonization and biocontrol activity of Bacillus amyloliquefaciens SQR9 by abrB gene disruption. Appl. Microbiol. Biotechnol. 97, 8823–8830. 10.1007/s00253-012-4572-4 23196984

[B49] Xu S. Liu Y.-X. Cernava T. Wang H. Zhou Y. Xia T.. (2022). Fusarium fruiting body microbiome member Pantoea agglomerans inhibits fungal pathogenesis by targeting lipid rafts. Nat. Microbiol. 7, 831–843. 10.1038/s41564-022-01131-x 35618775

[B50] Xue Q.-Y. Chen Y. Li S.-M. Chen L.-F. Ding G.-C. Guo D.-W.. (2009). Evaluation of the strains of Acinetobacter and Enterobacter as potential biocontrol agents against Ralstonia wilt of tomato. Biol. Control 48, 252–258. 10.1016/j.biocontrol.2008.11.004

[B51] Yang P. Zhao Z. Fan J. Liang Y. Bernier M. Gao Y.. (2023). Bacillus proteolyticus OSUB18 triggers induced systemic resistance against bacterial and fungal pathogens in Arabidopsis. Front. Plant Sci. 14, 1078100. 10.3389/fpls.2023.1078100 36755698 PMC9900001

[B52] Zapata A. Ramirez-Arcos S. (2015). A comparative study of McFarland turbidity standards and the Densimat photometer to determine bacterial cell density. Curr. Microbiol. 70, 907–909. 10.1007/s00284-015-0801-2 25794482

