# Supplementary material for: Enhancing plant defense using rhizobacteria in processing tomatoes: a bioprospecting approach to overcoming Early Blight and Alternaria toxins
Source: Front Microbiol. 2023 Aug 4;14:1221633. doi: 10.3389/fmicb.2023.1221633 (PMC10436473; doi:10.3389/fmicb.2023.1221633)
Supplement: Supplementary file 1 [file Data_Sheet_1.docx]

Supplementary Material

Enhancing plant defense with rhizobacteria in processing tomatoes: a bioprospecting approach to overcome Early Blight and Alternaria toxins

Gabriele Bellotti^1^, Maria Chiara Guerrieri^1^, Paola Giorni^2^, Giulia Bulla^2^, Andrea Fiorini^2^, Terenzio Bertuzzi^3^, Maria Elena Antinori^1*^, Edoardo Puglisi^1^.

^1^ Department for Sustainable Food Process, Università Cattolica del Sacro Cuore, Via Emilia Parmense 84, 29122, Piacenza,

^2^ Department of Sustainable Crop Production, Università Cattolica del Sacro Cuore, Via Emilia Parmense 84, 29122, Piacenza, Italy

^3^ Department of Animal Science, Food and Nutrition, Università Cattolica del Sacro Cuore, Via Emilia Parmense 84, 29122, Piacenza, Italy

Corresponding Author
mariaelena.antinori@unicatt.it

# Supplementary tables

**Table S1:** Ranking class system used to assign a score to each isolate for each parameter tested during the dual plate assay (fungal growth, increment of AOH, AME, TEN, TeA. More weight was assigned when isolates incremented the mycotoxins.

| **Reduction of fungal growth and mycotoxins production (%)** | **Rank score** |
| --- | --- |
| 0 | 0 |
| 0-25 | 0.5 |
| 26-50 | 1 |
| 51-75 | 1.5 |
| 76-100 | 2 |
| **Increment of mycotoxin production (%)** | **Rank score** |
| 0-25 (Small) | -1 |
| 26-50 (Medium) | -2 |
| 51-100 (High) | -3 |

**Table S2:** Taxonomic identification of unique bacterial strains isolated from the rhizosphere (80) and endosphere (13) of *Solanum lycopersicum* L. cultivated in crops managed with reduced tillage. Taxon identification is based on BLASTN of the 16S rDNA gene sequence on RDP database and NCBI. RDP was set with the following parameters: both, isolates, both, good, NCBI. The threshold for species assignment was set at 97.5% identity. Sequences are deposited at the accession number to be assigned. TR = Tomato Rhizosphere; TE= Tomato Endosphere. The datasets presented in this study can be found in online repositories. The names of the repository and accession numbers can be found at GenBank: OQ990921-OQ991008. P = pathogen, OP = opportunistic pathogen, NP = non pathogen.

| **Rhizosphere isolates** | | | | |
| --- | --- | --- | --- | --- |
| **Strain** | **Taxonomy** | **RDP S_ab Score/NCBI %ID** | **Pathogen** | **References** |
| TR1 | *Streptomyces violaceoruber* | 1.000 | NP |  |
| TR3 | *Variovorax paradoxus* | 0.975 | NP |  |
| TR4 | *Rhodococcus qingshengii* | 1.000 | NP |  |
| TR5 | *Rhodanobacter lindaniclasticus* | 0.988 | NP |  |
| TR6 | *Rhodanobacter soli* | 0.986 | NP |  |
| TR7 | *Microbacterium oxydans* | 0.998 | NP |  |
| TR8 | *Streptomyces dioscori* | 98.84 | NP |  |
| TR9 | *Rhodanobacter lindaniclasticus* | 0.988 | NP |  |
| TR10 | *Leifsonia shinshuensis* | 0.984 | NP |  |
| TR11 | *Priestia megaterium* | 1.000 | NP |  |
| TR12 | *Variovorax paradoxus* | 0.996 | NP |  |
| TR13 | *Leifsonia xyli* | 1.000 | NP |  |
| TR14 | *Microbacterium trichothecenolyticum* | 98.73 | NP |  |
| TR16 | *Stenotrophomonas maltophilia* | 0.980 | OP | (Brooke, 2012) |
| TR18 | *Paenibacillus panacihumi* | 0.975 | NP |  |
| TR17 | *Arthrobacter nitroguajacolicus* | 0.989 | NP |  |
| TR18 | *Paenibacillus panacihumi* | 98.45 | NP |  |
| TR19 | *Terribacillus goriensis* | 99.47 | NP |  |
| TR20 | *Terribacillus saccharophilus* | 1.000 | NP |  |
| TR21 | *Microbacterium phyllosphaerae* | 1.000 | NP |  |
| TR22 | *Solibacillus silvestris* | 1.000 | NP |  |
| TR23 | *Bacillus humi* | 0.976 | NP |  |
| TR25 | *Bosea thiooxidans* | 98.77 | NP |  |
| TR26 | *Chitinophaga longshanensis* | 98.90 | NP |  |
| TR27 | *Streptomyces clavuligerus* | 97.54 | NP |  |
| TR28 | *Paenibacillus amylolyticus* | 1.000 | NP |  |
| TR29 | *Chryseobacterium cheonjiense* | 99.83 | NP |  |
| TR30 | *Pseudomonas fluorescens* | 1.000 | NP |  |
| TR31 | *Chryseobacterium ureilyticum* | 1.000 | NP |  |
| TR33 | *Stenotrophomonas maltophilia* | 1.000 | NP |  |
| TR34 | *Sphingobacterium athyrii* | 98.930 | NP |  |
| TR35 | *Bacillus cereus sl* | 99.240 | P | (Tuipulotu et al., 2021) |
| TR36 | *Bacillus subtilis* | 1.000 | NP |  |
| TR37 | *Bacillus cereus sl* | 0.992 | P | (Tuipulotu et al., 2021) |
| TR38 | *Bacillus pumilus* | 1.000 | NP |  |
| TR40 | *Serratia nematodiphila* | 1.000 | NP |  |
| TR41 | *Paraburkholderia graminis* | 99.290 | NP |  |
| TR43 | *Ralstonia pickettii* | 100.000 | NP |  |
| TR44 | *Streptomyces rishiriensis* | 99.610 | NP |  |
| TR45 | *Rhodanobacter spathiphylli* | 99.390 | NP |  |
| TR46 | *Stenotrophomonas rhizophila* | 99.510 | NP |  |
| TR48 | *Rhodanobacter xiangquanii* | 98.810 | NP |  |
| TR49 | *Chryseobacterium soli* | 98.640 | NP |  |
| TR50 | *Bacillus frigoritolerans* | 98.550 | NP |  |
| TR51 | *Bacillus pumilus* | 1.000 | NP |  |
| TR52 | *Microbacterium oleivorans* | 1.000 | NP |  |
| TR53 | *Bacillus cereus sl* | 0.980 | P | (Tuipulotu et al., 2021) |
| TR54 | *Chryseobacterium soli* | 98.910 | NP |  |
| TR55 | *Stenotrophomonas maltophilia* | 1.000 | NP |  |
| TR56 | *Pseudomonas thivervalensis* | 0.988 | NP |  |
| TR57 | *Bacillus safensis* | 99.610 | NP |  |
| TR58 | *Paenibacillus amylolyticus* | 1.000 | NP |  |
| TR59 | *Bacillus pumilus* | 1.000 | NP |  |
| TR60 | *Pseudomonas koreensis* | 1.000 | NP |  |
| TR61 | *Enterobacter asburiae* | 0.994 | OP | (Mustafa et al., 2020) |
| TR62 | *Bacillus subtilis* | 1.000 | NP |  |
| TR63 | *Streptomyces globisporus* | 99.410 | NP |  |
| TR64 | *Delftia acidovorans* | 0.988 | NP |  |
| TR65 | *Variovorax boronicumulans* | 0.982 | NP |  |
| TR66 | *Streptomyces griseoaurantiacus* | 0.980 | NP |  |
| TR68 | *Terrabacter tumescens* | 1.000 | NP |  |
| TR69 | *Rhodanobacter lindaniclasticus* | 99.130 | NP |  |
| TR71 | *Leifsonia shinshuensis* | 0.991 | NP |  |
| TR72 | *Stenotrophomonas maltophilia* | 0.988 | NP |  |
| TR73 | *Rhizobium radiobacter* | 0.988 | P | (White & Winans, 2007) |
| TR74 | *Priestia megaterium* | 0.995 | NP |  |
| TR75 | *Brevundimonas terrae* | 0.978 | NP |  |
| TR78 | *Tsukamurella pulmonis* | 98.710 | P | (Yassin et al., 1996) |
| TR81 | *Xanthomonas axonopodis* | 0.983 | P |  |
| TR82 | *Stenotrophomonas maltophilia* | 1.000 | NP |  |
| TR84 | *Chitinophaga polysaccharea* | 98.710 | NP |  |
| TR85 | *Terrabacter tumescens* | 99.200 | NP |  |
| TR86 | *Chryseobacterium soli* | 98.750 | NP |  |
| TR87 | *Xanthomonas hydrangeae* | 1.000 | P | (Mhedbi-Hajri et al., 2013) |
| TR88 | *Pseudomonas brassicacearum* | 0.990 | OP | (Gislason & de Kievit, 2020) |
| TR89 | *Stenotrophomonas chelatiphaga* | 1.000 | NP |  |
| TR91 | *Luteibacter rhizovicinus* | 98.430 | NP |  |
| TR92 | *Bacillus subtilis* | 1.000 | NP |  |
| TR93 | *Priestia megaterium* | 1.000 | NP |  |
| TR94 | *Acinetobacter calcoaceticus* | 0.993 | NP |  |
| **Endosphere isolates** | | | | |
| **Strain** | **Taxonomy** | **RDP S_ab Score/NCBI %ID** | **Pathogen** | **References** |
| TE95 | *Kluyvera cryocrescens* | 99.140 | OP | (Wong, 1987) |
| TE98 | *Serratia nematodiphila* | 99.390 | NP |  |
| TE99 | *Enterobacter ludwigii* | 1.000 | OP | (Mustafa et al., 2020) |
| TE100 | *Enterobacter aerogenes* | 0.992 | NP |  |
| TE103 | *Enterobacter asburiae* | 0.990 | OP | (Mustafa et al., 2020) |
| TE105 | *Pseudomonas citronellolis* | 1.000 | NP |  |
| TE106 | *Bacillus amyloliquefaciens* | 1.000 | NP |  |
| TE108 | *Kosakonia cowanii* | 0.992 | NP |  |
| TE109 | *Pantoea agglomerans* | 0.995 | OP | (Dutkiewicz et al., 2016) |
| TE110 | *Serratia nematodiphila* | 1.000 | NP |  |
| TE114 | *Pseudomonas nitroreducens* | 1.000 | NP |  |
| TE116 | *Pantoea agglomerans* | 0.995 | OP | (Dutkiewicz et al., 2016) |
| TE117 | *Serratia marcescens* | 1.000 | NP |  |

**Table S3:** Dual plate assay second screening. Results are expressed in % of difference between the fungal biomass and Alternaria toxin values of *A. alternata*, *A. tenuissima* and *A. solani* when inoculated alone on PDA and when co-inoculated in the dual plate assay incubated for 14 days. TeA= tenuazonic acid; AOH= alternariol; AME= alternariol monomethyl ether; TEN= tentoxin. NG = No growth.

|  |  | | ***A. alternata*** | | | | | ***A. solani*** | | | | ***A. tenuissima*** | | | | |
| --- | --- | --- | --- | --- | --- | --- | --- | --- | --- | --- | --- | --- | --- | --- | --- | --- |
| **Strain** | | **Taxonomy** | **Fungal biomass** | **TeA** | **AOH** | **AME** | **TEN** | **Fungal biomass** | **AOH** | **AME** | **TEN** | **Fungal biomass** | **TeA** | **AOH** | **AME** | **TEN** |
| TR1 | *Streptomyces violaceoruber* | | -18.2 | -49.8 | 0.8 | 34.4 | -51.5 | -5.9 | 379.8 | 365.1 | 177.2 | 0.0 | -3.4 | 168.4 | 251.0 | -35.7 |
| TR3 | *Variovorax paradoxus* | | -33.0 | -77.9 | -58.9 | -73.1 | 198.3 | 8.8 | 43.9 | -19.6 | 2161.6 | -15.7 | -4.2 | 718.2 | 757.3 | 927.5 |
| TR4 | *Rhodococcus qingshengii* | | 1.0 | -64.3 | 45.6 | 86.4 | -94.4 | -24.9 | 89.7 | -26.8 | 1061.5 | 0.0 | 38.1 | -18.9 | -80.1 | -72.2 |
| TR8 | *Streptomyces dioscori* | | -5.5 | -25.5 | 74.6 | 54.8 | -91.0 | -6.5 | 36.2 | 29.1 | 5996.4 | 0.0 | 45.5 | 284.2 | 476.6 | -14.1 |
| TR10 | *Leifsonia shinshuensis* | | -4.2 | -54.3 | 6.3 | -2.1 | -88.2 | -3.1 | -44.1 | -11.1 | 25442.3 | 0.0 | 22.5 | 279.0 | 275.2 | 26.9 |
| TR11 | *Priestia megaterium* | | -4.8 | 6.4 | 56.7 | 93.3 | -48.6 | 13.6 | -4.3 | 1.2 | 45719.1 | 0.0 | 12.7 | 128.0 | 300.4 | 235.8 |
| TR13 | *Leifsonia xyli* | | -13.9 | -72.3 | -60.7 | -63.1 | -60.9 | 3.8 | -68.1 | -59.3 | 83112.3 | 0.0 | 13.2 | 327.6 | 356.0 | -52.3 |
| TR14 | *Microbacterium trichothecenolyticum* | | -17.2 | -42.4 | -36.9 | -31.7 | -41.3 | 7.1 | -52.2 | 11.9 | 18104.3 | 0.0 | 27.7 | 104.5 | 260.4 | -15.1 |
| TR17 | *Arthrobacter nitroguajacolicus* | | 3.2 | -94.9 | 2.0 | 59.3 | -78.8 | 2.2 | 41.4 | 20.8 | 9279.2 | 0.0 | -98.1 | 316.2 | 228.8 | -90.8 |
| TR18 | *Paenibacillus panacihumi* | | -20.6 | -13.0 | 80.5 | 27.1 | -80.7 | 4.2 | 490.1 | 683.9 | 14958.3 | NG | - | - | - | - |
| TR27 | *Streptomyces clavuligerus* | | -5.6 | -6.9 | 53.5 | 36.1 | -64.5 | 5.1 | 29.8 | 48.7 | 930.6 | 0.0 | -9.8 | 58.0 | 98.5 | -15.8 |
| TR30 | *Pseudomonas fluorescens* | | -20.5 | -81.4 | -11.7 | -53.7 | -46.2 | -28.7 | -41.2 | -46.6 | 700.2 | -14.1 | -34.1 | 157.5 | 124.7 | 289.9 |
| TR31 | *Chryseobacterium ureilyticum* | | -16.8 | -78.2 | -65.6 | -57.0 | -64.0 | 14.7 | -46.8 | 8.0 | 130612.8 | -11.1 | -57.6 | 353.5 | 449.6 | -0.8 |
| TR38 | *Bacillus pumilus* | | -23.2 | -53.2 | 42.6 | -39.2 | 198.9 | -10.9 | 21.1 | 117.7 | 62094.7 | 0.0 | 35.0 | 6.2 | 186.1 | 18.2 |
| TR40 | *Serratia nematodiphila* | | -25.6 | -85.0 | -70.8 | -92.8 | 52.1 | -14.5 | -84.0 | -84.8 | -5.2 | -32.1 | -81.0 | -88.2 | -70.0 | -70.6 |
| TR52 | *Microbacterium oleivorans* | | -12.9 | -16.5 | 55.8 | 71.6 | -69.7 | -11.4 | 376.9 | 165.6 | 7120.8 | 0.0 | 105.2 | 307.4 | 391.2 | 65.5 |
| TR54 | *Chryseobacterium soli* | | NG | - | - | - | - | 27.0 | 398.0 | 577.7 | 21888.2 | -16.9 | -4.4 | -34.9 | 19.6 | -27.6 |
| TR55 | *Stenotrophomonas maltophilia* | | 2.7 | -69.0 | -22.4 | -61.8 | -57.1 | 34.1 | 382.8 | 253.8 | 117300.8 | 0.0 | 37.1 | 291.2 | 338.6 | -2.0 |
| TR56 | *Pseudomonas thivervalensis* | | -18.8 | -67.0 | -48.0 | 38.0 | -92.0 | -20.2 | 2004.1 | 955.6 | 10803.5 | -35.0 | 44.2 | 188.2 | 1226.5 | -44.4 |
| TR57 | *Bacillus safensis* | | -28.7 | -77.1 | -11.7 | 23.4 | 582.9 | -41.9 | 11.1 | 136.4 | 34825.3 | -40.0 | -37.7 | 180.6 | 1375.6 | 181.8 |
| TR58 | *Paenibacillus amylolyticus* | | -13.4 | -9.5 | 126.5 | 109.5 | -71.6 | 11.1 | 334.8 | 253.3 | 4127.7 | NG | - | - | - | - |
| TR59 | *Bacillus pumilus* | | -14.8 | -45.9 | 34.8 | 6.5 | -62.5 | 11.4 | -67.7 | -38.8 | 124142.6 | -16.9 | 82.8 | -57.1 | -8.7 | 82.2 |
| TR60 | *Pseudomonas koreensis* | | 7.1 | -93.6 | -87.3 | -66.0 | -92.1 | -26.7 | -53.3 | -34.9 | 260.9 | -26.3 | 11.4 | 474.2 | 607.3 | 662.9 |
| TR61 | *Enterobacter asburiae* | | -14.0 | -65.4 | -19.8 | 2.4 | -84.1 | -0.9 | 1259.1 | 727.0 | 10318.1 | -25.1 | 94.4 | 21.4 | 150.7 | 241.6 |
| TR62 | *Bacillus subtilis* | | -67.6 | -99.5 | -52.7 | -90.0 | -98.1 | -57.0 | 58.4 | -41.7 | 85.8 | -76.7 | -95.4 | 284.5 | 4844.4 | -99.9 |
| TR65 | *Variovorax boronicumulans* | | 12.2 | -98.7 | -40.5 | -87.1 | 409.6 | 22.9 | -27.2 | -47.5 | 1651.0 | 0.0 | -98.4 | 537.1 | 301.7 | 52.1 |
| TR66 | *Streptomyces griseoaurantiacus* | | -23.8 | -53.4 | 54.6 | 53.0 | -81.1 | -4.2 | 147.5 | 123.8 | 1735.6 | 0.0 | 52.8 | 159.5 | 161.1 | 41.3 |
| TR72 | *Stenotrophomonas maltophilia* | | -2.2 | -37.2 | 76.2 | 100.1 | -38.0 | 38.8 | 139.0 | 42.6 | 1485.4 | 0.0 | 62.8 | 503.8 | 582.9 | 10.8 |
| TR82 | *Stenotrophomonas maltophilia* | | 21.0 | -79.4 | -56.8 | -79.8 | -46.7 | 55.5 | 27.3 | 32.4 | 11697.1 | 0.0 | 25.1 | 754.9 | 604.8 | 65.2 |
| TR84 | *Chitinophaga polysaccharea* | | -11.4 | -67.7 | -8.9 | -61.5 | -37.5 | 10.7 | -71.3 | -45.0 | 65170.3 | 0.0 | -46.3 | -6.4 | 19.0 | 1018.7 |
| TR88 | *Pseudomonas brassicacearum* | | -9.2 | -47.0 | -31.0 | 123.8 | -26.2 | 15.8 | 161.2 | 428.9 | 1001.9 | -21.6 | 90.7 | -60.9 | -13.7 | -56.2 |
| TR91 | *Luteibacter rhizovicinus* | | -25.3 | -71.6 | -22.1 | 49.6 | 101.6 | -13.7 | 127.8 | 189.2 | 155.2 | -59.7 | -34.9 | -72.4 | -67.6 | 280.7 |
| TR92 | *Bacillus subtilis* | | -64.1 | -99.7 | -47.6 | -78.1 | -68.8 | -65.0 | 19.2 | -27.3 | -44.2 | -76.0 | -97.7 | 82.3 | 2842.8 | -99.9 |
| TR93 | *Priestia megaterium* | | -9.5 | -51.4 | 15.5 | 9.8 | -76.9 | 21.6 | 23.4 | 42.8 | 87316.3 | 0.0 | 26.9 | 48.8 | 165.6 | 269.4 |
| TE98 | *Serratia nematodiphila* | | -26.9 | -86.5 | -57.3 | -89.8 | -26.6 | -32.1 | -94.6 | -97.4 | -91.3 | -28.8 | -80.6 | -89.5 | 39.9 | -44.4 |
| TE99 | *Enterobacter ludwigii* | | -26.6 | -80.8 | -41.1 | -77.2 | 4.3 | -26.5 | -88.1 | -92.7 | -26.2 | -12.4 | -76.8 | -93.8 | -73.5 | -41.2 |
| TE103 | *Enterobacter asburiae* | | -20.9 | -75.9 | -31.6 | -83.2 | 49.0 | -15.4 | -87.1 | -96.2 | -58.2 | -10.4 | -70.4 | -79.9 | -52.0 | 5.3 |
| TE105 | *Pseudomonas citronellolis* | | -21.4 | -54.7 | 45.2 | 141.2 | -69.0 | 4.7 | -34.1 | -21.3 | 72343.9 | NG | - | - | - | - |
| TE106 | *Bacillus amyloliquefaciens* | | -60.9 | -99.6 | -42.7 | -70.8 | -88.2 | -71.7 | -34.5 | -51.7 | 2985.8 | -73.6 | -96.4 | 215.0 | 3501.2 | -99.6 |
| TE108 | *Kosakonia cowanii* | | -24.5 | -77.1 | -30.5 | -64.4 | -32.0 | -3.6 | -87.7 | -89.9 | 4784.7 | -7.2 | -52.8 | -88.7 | -52.3 | 543.1 |
| TE109 | *Pantoea agglomerans* | | -21.3 | -78.1 | -29.0 | -82.1 | 7.0 | -29.0 | -78.5 | -87.5 | 5008.5 | -16.0 | -80.8 | -64.3 | -20.0 | -24.5 |
| TE110 | *Serratia nematodiphila* | | -24.0 | -85.2 | -46.5 | -91.8 | -55.4 | -25.2 | -91.8 | -97.4 | 6576.7 | -25.3 | -64.3 | -81.9 | -73.7 | -56.4 |
| TE114 | *Pseudomonas nitroreducens* | | -24.8 | -36.0 | 254.7 | 352.4 | -58.7 | 6.5 | -71.9 | -34.9 | 235535.0 | -11.1 | 44.4 | 275.1 | 247.6 | 46.6 |
| TE116 | *Pantoea agglomerans* | | -23.2 | -75.7 | -7.1 | -77.9 | -30.0 | -24.9 | -66.0 | -81.8 | 1843.0 | -16.3 | -72.8 | -71.9 | -37.4 | 29.2 |
| TE117 | *Serratia marcescens* | | -23.4 | -82.3 | -36.9 | -92.1 | 24.5 | -18.0 | -91.5 | -95.8 | 11974.2 | -35.7 | -78.8 | -87.4 | -29.3 | -12.7 |

# Supplementary Figures

**
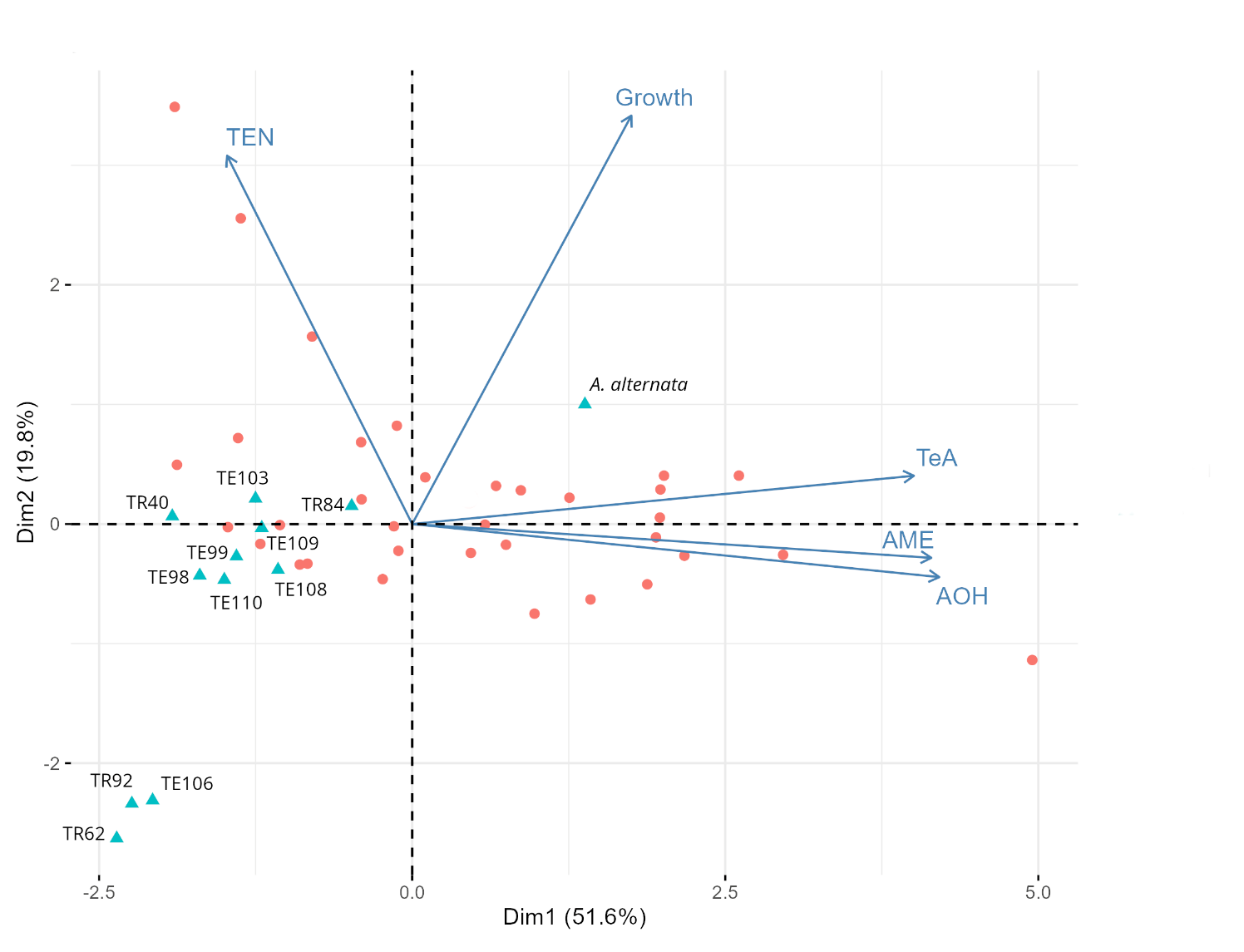
**

**Supplementary Figure 1:** Principal component analysis (PCA) among isolates on *A. alternata* growth diameter (Growth) and on its production of mycotoxins tenuazonic acid (TeA), alternariol (AOH), alternariol monomethyl ether (AME) and tentoxin (TEN). Average points are represented (n=5 for growth, n=3 for mycotoxins). Top 12 strains of the ranking and the reference are represented by light-blue triangles, while the other strains are represented by red circles.


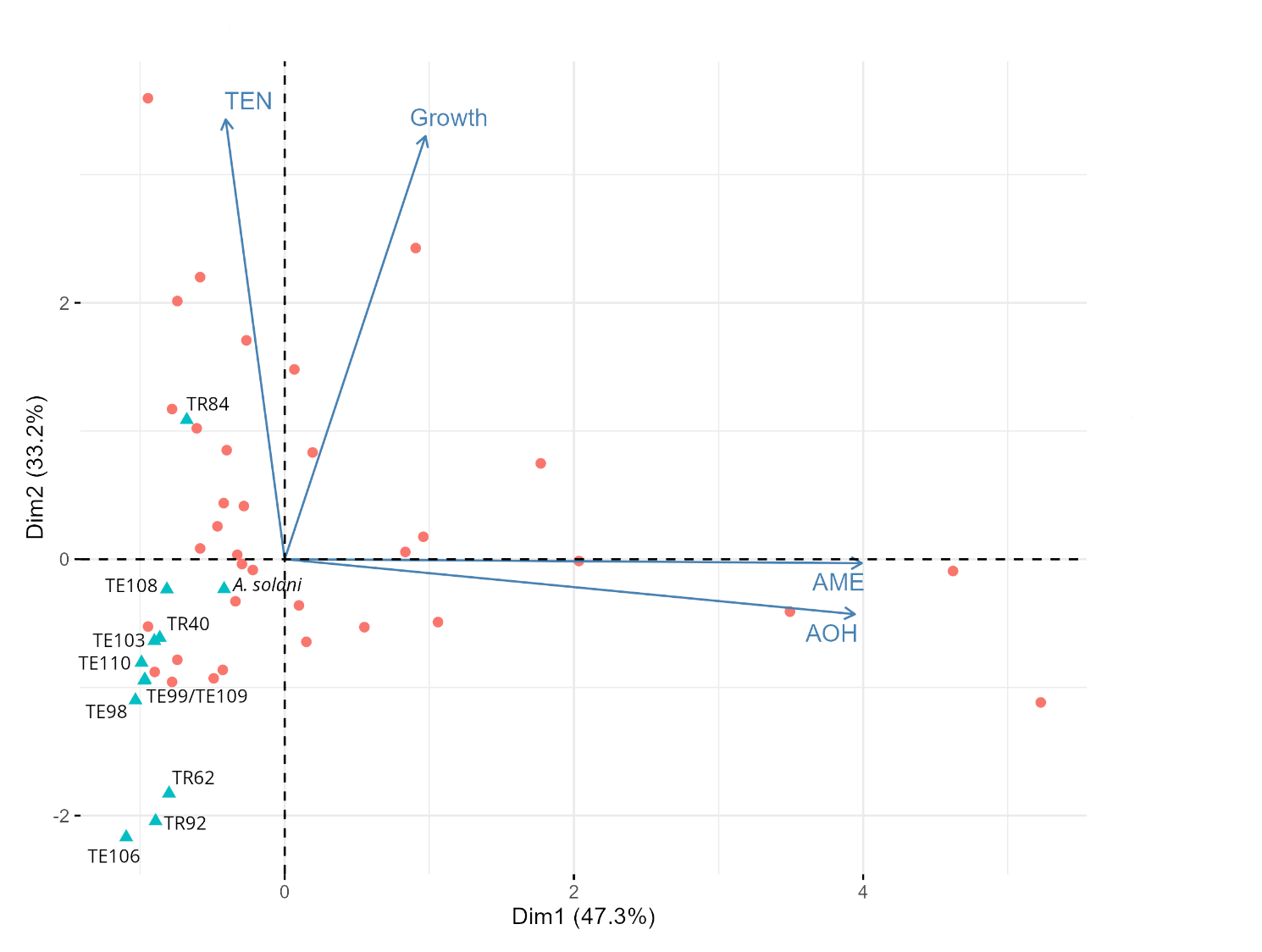


**Supplementary Figure 2:** Principal component analysis (PCA) among isolates on *A. solani* growth diameter (Growth) and on its production of mycotoxins tenuazonic acid (TeA), alternariol (AOH), alternariol monomethyl ether (AME) and tentoxin (TEN). Average points are represented (n=5 for growth, n=3 for mycotoxins). Top 12 strains of the ranking and the reference are represented by light-blue triangles, while the other strains are represented by red circles.


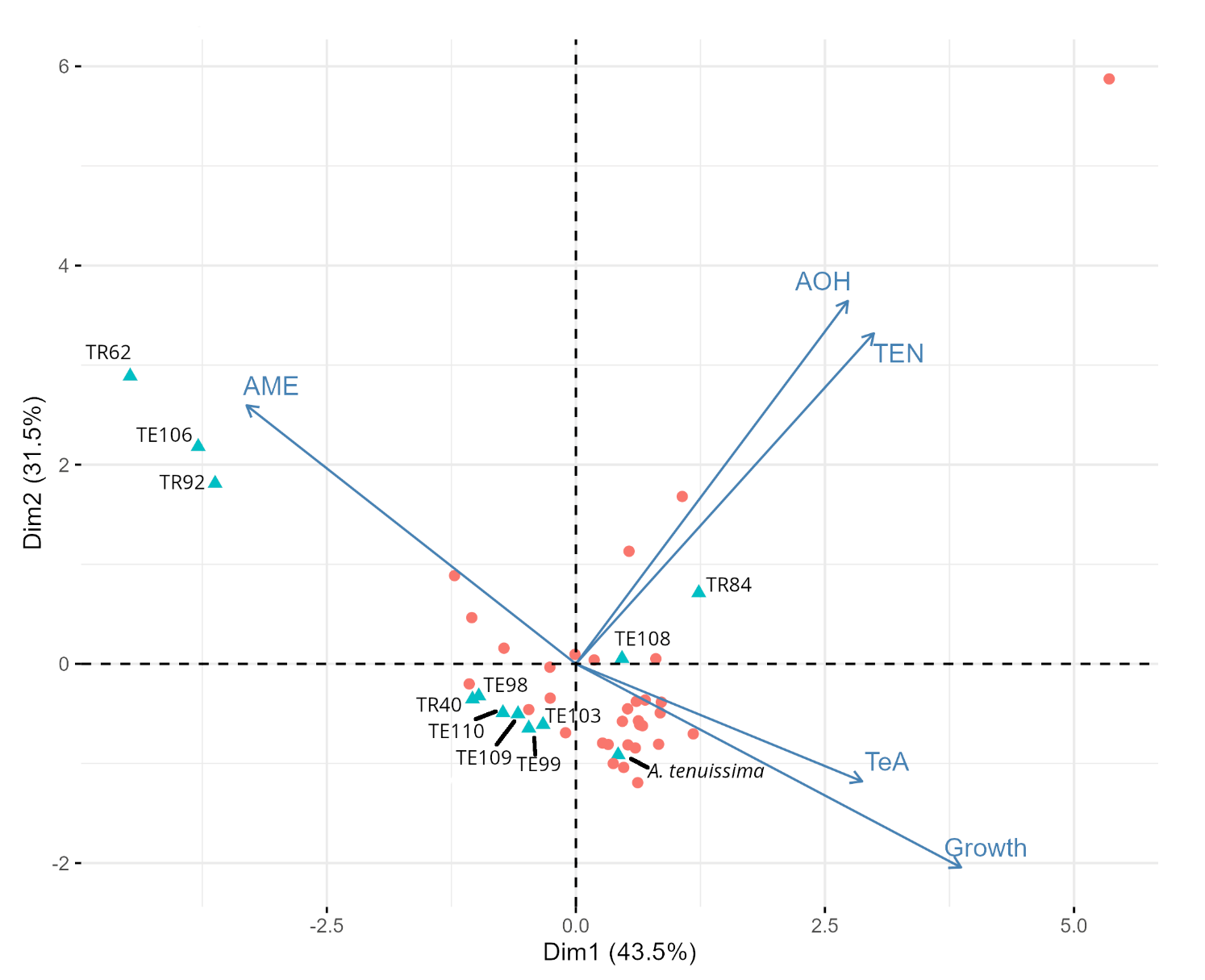


**Supplementary Figure 3:** Principal component analysis (PCA) among isolates on *A. tenuissima* growth diameter (Growth) and on its production of mycotoxins tenuazonic acid (TeA), alternariol (AOH), alternariol monomethyl ether (AME) and tentoxin (TEN). Average points are represented (n=5 for growth, n=3 for mycotoxins). Top 12 strains of the ranking and the reference are represented by light-blue triangles, while the other strains are represented by red circles.

**References**

Brooke, J. S. (2012). Stenotrophomonas maltophilia: an emerging global opportunistic pathogen. *Clinical Microbiology Reviews*, *25*(1), 2–41.

Dutkiewicz, J., Mackiewicz, B., Lemieszek, M. K., Golec, M., & Milanowski, J. (2016). Pantoea agglomerans: a mysterious bacterium of evil and good. Part IV. Beneficial effects. *Annals of Agricultural and Environmental Medicine*, *23*(2).

Gislason, A. S., & de Kievit, T. R. (2020). Friend or foe? Exploring the fine line between Pseudomonas brassicacearum and phytopathogens. *Journal of Medical Microbiology*, *69*(3), 347–360.

Mhedbi-Hajri, N., Hajri, A., Boureau, T., Darrasse, A., Durand, K., Brin, C., Saux, M. F.-L., Manceau, C., Poussier, S., & Pruvost, O. (2013). Evolutionary history of the plant pathogenic bacterium Xanthomonas axonopodis. *Plos One*, *8*(3), e58474.

Mustafa, A., Ibrahim, M., Rasheed, M. A., Kanwal, S., Hussain, A., Sami, A., Ahmed, R., & Bo, Z. (2020). Genome-wide Analysis of Four Enterobacter cloacae complex type strains: Insights into Virulence and Niche Adaptation. *Scientific Reports*, *10*(1), 8150.

Tuipulotu, D. E., Mathur, A., Ngo, C., & Man, S. M. (2021). Bacillus cereus: epidemiology, virulence factors, and host–pathogen interactions. *Trends in Microbiology*, *29*(5), 458–471.

White, C. E., & Winans, S. C. (2007). Cell–cell communication in the plant pathogen Agrobacterium tumefaciens. *Philosophical Transactions of the Royal Society B: Biological Sciences*, *362*(1483), 1135–1148.

Wong, V. K. (1987). Broviac catheter infection with Kluyvera cryocrescens: a case report. *Journal of Clinical Microbiology*, *25*(6), 1115–1116.

Yassin, A. F., Rainey, F. A., Brzezinka, H., Burghardt, J., Rifai, M., Seifert, P., Feldmann, K., & Schaal, K. P. (1996). Tsukamurella pulmonis sp. nov. *International Journal of Systematic and Evolutionary Microbiology*, *46*(2), 429–436.
